# Purinergic Receptor Nanoimmunoamplifiers Potentiate Chemoimmunotherapy Efficacy in Hepatocellular Carcinoma

**DOI:** 10.34133/bmr.0278

**Published:** 2025-11-06

**Authors:** Jialiang Zhang, Jinyu Zhang, Qiang Feng, Xin Jiang, Yong Yang, Wenhan Liu, Jianbin Xiao, Jukai Feng, Zhiyu Wang, Meiqi Pan, Jianmin Wang, Jingfeng Liu

**Affiliations:** ^1^ Innovation Center for Cancer Research, Clinical Oncology School of Fujian Medical University, Fujian Cancer Hospital, Fuzhou 350014, PR China.; ^2^ Fujian Key Laboratory of Advanced Technology for Cancer Screening and Early Diagnosis, Clinical Oncology School of Fujian Medical University, Fujian Cancer Hospital, Fuzhou 350014, PR China.; ^3^ Department of Hepatopancreatobiliary Surgery, Fujian Cancer Hospital, Clinical Oncology School of Fujian Medical University, Fuzhou 350014, PR China.

## Abstract

The effectiveness of chemoimmunotherapy for hepatocellular carcinoma (HCC) is hindered by the weak immunogenicity of chemotherapy-induced immunogenic cell death (ICD). This limitation primarily stems from the insufficient activation of the extracellular adenosine triphosphate (eATP)/P2X7 purinergic receptor (P2X7R)/NOD-, LRR-, and pyrin domain-containing protein 3 (NLRP3) inflammasome pathway in dendritic cells (DCs). To address this challenge, we designed ivermectin–MnO_2_ nanocomplexes (IMNs) as P2X7R-targeted nanoimmunoamplifiers to enhance the immunogenicity of chemotherapy-induced ICD. The ivermectin component of IMN enhanced liposomal doxorubicin (LD)-induced ICD and increased P2X7R sensitivity to eATP. Additionally, the MnO_2_ component of IMN alleviated tumor hypoxia and down-regulated CD39/CD73 expression, thereby preventing eATP degradation. These combined strategies robustly activated the eATP/P2X7R/NLRP3 inflammasome cascade in DCs, eliciting a potent antitumor immune response. In combination with anti-PD-L1 antibody and LD, IMN effectively inhibited tumor growth in orthotopic, subcutaneous, and metastatic HCC mouse models. Our study underscores the crucial role of IMN in amplifying the NLRP3 inflammasome cascade in DCs during ICD, presenting a promising strategy to enhance the efficacy of HCC chemoimmunotherapy.

## Introduction

Hepatocellular carcinoma (HCC) remains a leading cause of cancer deaths, with a rising incidence that underscores significant therapeutic challenges [1]. Despite the transformative impact of immune checkpoint inhibitors (ICIs) on HCC treatment, the current standard atezolizumab–bevacizumab regimen achieves an objective response rate of only 30%. This highlights the need for enhanced therapeutic strategies [2]. Chemoimmunotherapy, which combines chemotherapy with ICIs, operates through a distinct mechanism and holds promise for improving responses in the 70% of HCC patients who do not respond to atezolizumab–bevacizumab therapy [3]. However, common HCC chemotherapies, such as transarterial chemoembolization (TACE) and hepatic arterial infusion chemotherapy (HAIC), fail to optimally enhance ICI responses. This is primarily due to inadequate immunogenicity of chemotherapy-induced immunogenic tumor cell death (ICD) [4,5]. Extracellular adenosine triphosphate (eATP) is a key endogenous damage-associated molecular pattern (DAMP) released during chemotherapy-induced ICD. It engages with P2X7 purinergic receptors (P2X7R) on dendritic cells (DCs) to prime the NOD-, LRR-, and pyrin domain-containing protein 3 (NLRP3) inflammasome cascade. This cascade is essential for the immunostimulatory effects of chemotherapy-induced ICD. It leads to interleukin-1β (IL-1β) secretion and activation of tumor antigen-specific, interferon γ (IFNγ)-producing CD8^+^ T cells [6]. Reduced eATP levels, resulting from impaired autophagy during chemotherapy, critically undermine immunotherapeutic efficacy and nullify the antitumoral effects [7]. Therefore, sustaining sufficient eATP levels over time is essential to maintain the P2X7R/NLRP3 inflammasome cascade in DCs, posing a critical challenge for optimizing the immunogenic potential of chemotherapy.

Given ATP’s role as a ubiquitous energy source, it is challenging to selectively modulate eATP levels to sustain P2X7R/NLRP3 signaling and enhance chemotherapy-induced immunogenicity in the tumor microenvironment (TME). Notably, among P2X receptors, P2X7R activation requires the highest eATP concentrations (0.5 to 1.0 mM). Typically, the TME maintains eATP at concentrations ranging from 0.1 to 0.5 mM. Chemotherapy triggers a surge in eATP release but only transiently activates the P2X7R/NLRP3 inflammasome cascade [3,8]. To date, only one study has attempted to coadminister ATP with chemotherapy drugs to tumor sites. This approach preserves eATP levels during chemotherapy-induced ICD, improving chemoimmunotherapy outcomes. However, the immunomodulatory role of the P2X7R/NLRP3 pathway in this context remains unexplored [9]. Moreover, the clinical translation of direct ATP delivery was hindered by its intrinsic instability and propensity for rapid degradation into immunosuppressive extracellular adenosine (eADO) due to enzymatic degradation by CD39 and CD73 [10]. Therefore, it is urgently necessary to develop a method that can effectively and selectively modulate eATP levels to maintain P2X7R/NLRP3 signaling, thereby enhancing chemotherapy-mediated immunogenicity in the TME.

Another challenge arises in sustaining P2X7R/NLRP3 pathway activation through eATP modulation: The TME is ubiquitously hypoxic. Hypoxia triggers the stabilization of hypoxia-inducible factor-1α (HIF-1α), which in turn up-regulates the expression of CD39 and CD73 [11]. This up-regulation accelerates eATP degradation into immunosuppressive adenosine. Consequently, P2X7R/NLRP3 signaling efficacy is reduced, and the antitumor immune response is suppressed [10]. Systemic administration of high-concentration oxygen (>60% O_2_) can counteract hypoxia-induced adenosinergic immunosuppression. However, such high oxygen levels are associated with severe side effects, including pulmonary inflammation, alveolar infiltration, and fibrosis [12,13]. Conversely, lower oxygen concentrations may be safer but less effective against solid tumors distant from the lungs [13]. Therefore, there is a critical demand for innovative therapeutic strategies that effectively alleviate tumor hypoxia, reverse hypoxia–adenosinergic immunosuppression, and minimize off-target toxicities.

**Fig. 1.**
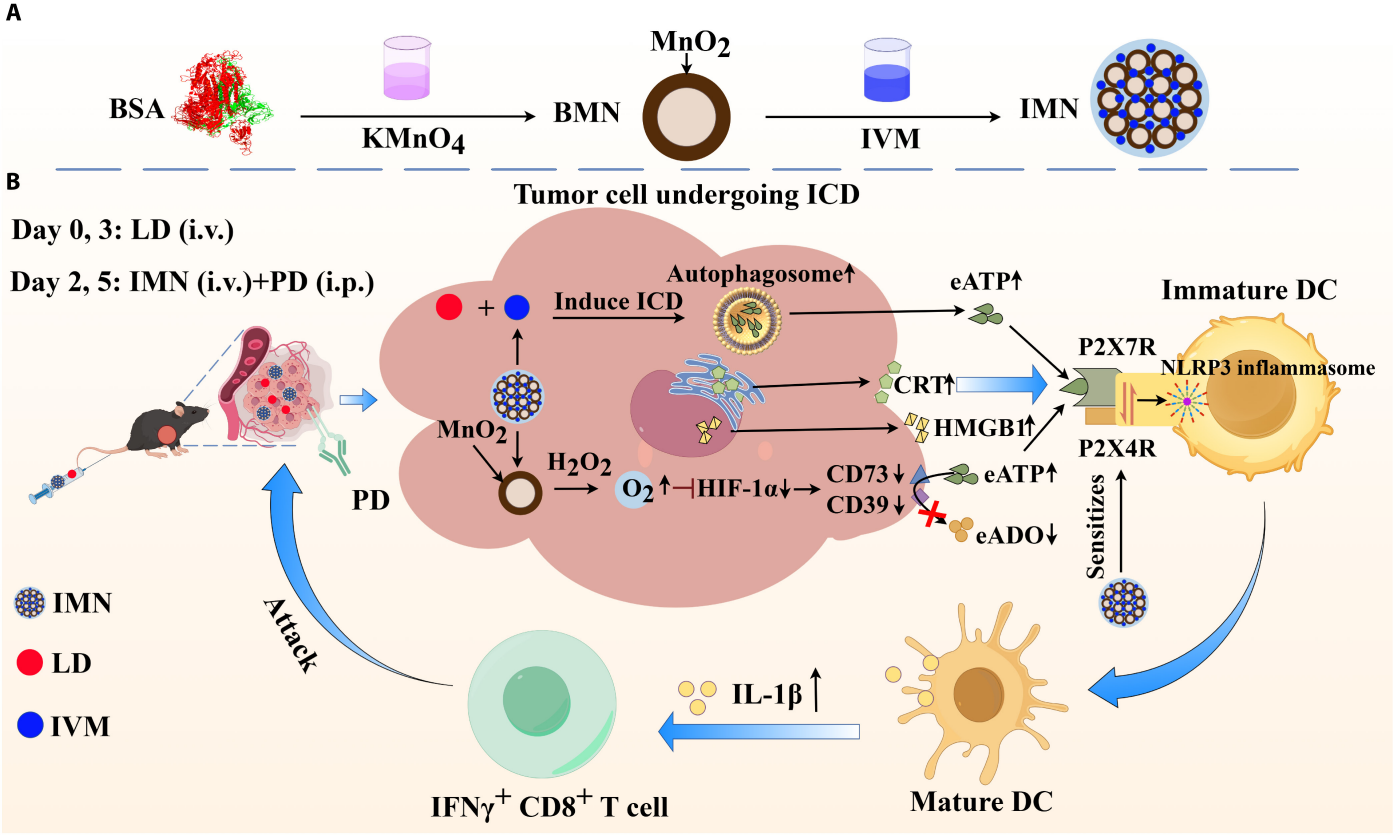
(A) Schematic illustration of the IMN synthesis, which begins with the reduction of KMnO_4_ mediated by the amino and sulfhydryl functionalities of BSA, yielding BMN. Subsequently, the assembly of IMN is accomplished by capitalizing on hydrophobic interactions to effectuate the binding between BMN and IVM. (B) A schematic depiction elucidates the synergistic effects where IMN potentiates LD-elicited ICD and eATP release while concurrently sensitizing P2X4R/P2X7R to eATP. Moreover, the MnO_2_ component of IMN alleviates tumor hypoxia and down-regulates CD39/CD73, thus curtailing eATP degradation. Collectively, these actions culminate in robust NLRP3 inflammasome activation in DCs, manifesting as augmented IL-1β secretion and augmented infiltration of IFNγ^+^ CD8^+^ T lymphocytes into tumors. The integration of IMN with a chemoimmunotherapy regimen, encompassing anti-programmed death-ligand 1 antibody (PD) and LD, effectively restrains tumor growth in murine models of HCC.

Based on the preceding discussion, we propose a novel synergistic strategy combining ivermectin (IVM) and MnO_2_ nanostructures. Here, IVM, which acts as a positive allosteric modulator of P2X4R, is a U.S. Food and Drug Administration (FDA)-approved antiparasitic drug that enhances NLRP3 inflammasome signaling, while MnO_2_ alleviates tumor hypoxia to preserve eATP levels [14]. An established interaction between P2X4R and P2X7R leads to the formation of functional signaling complexes. IVM sensitizes these complexes, enhancing their functional activity. This sensitization subsequently triggers NLRP3 inflammasome activation, a mechanism validated by extensive studies [15–17]. Additionally, IVM’s ability to induce both ICD and autophagy may enhance eATP release during chemotherapy [7,18,19]. To validate our hypothesis, we designed and synthesized IVM–MnO_2_ nanocomplexes (IMNs) to function as P2X7R nanoimmunoamplifiers through an optimized synthesis procedure. Bovine serum albumin (BSA)-stabilized MnO_2_ nanoparticles (BMNs) were synthesized by reducing KMnO_4_ using BSA’s amino and sulfhydryl groups. Following this, IMN was assembled by harnessing hydrophobic interactions to facilitate the binding of BMN to IVM via a nanoprecipitation method [20,21]. In a Hepa1-6 HCC xenograft mouse model, we integrated IMN into a chemoimmunotherapy regimen combining anti-programmed death-ligand 1 antibody (PD) and hydrochloride liposomes (LD). This combination significantly amplified eATP/P2X7R/NLRP3 inflammasome cascade activation in DCs. This led to a substantial increase in intratumoral and systemic IL-1β concentrations, enhancing tumor-specific IFNγ-mediated CD8^+^ T cell responses. Consequently, this synergistic strategy effectively curtailed the progression of orthotopic, subcutaneous, and metastatic tumors (Fig. 1).

## Materials and Methods

### Materials

BSA and adenosine 5′-triphosphate disodium salt hydrate (A2383) were obtained from Sigma-Aldrich (St. Louis, MO, USA). Potassium permanganate was procured from Sinopharm Chemical Reagent Co. Ltd. (Shanghai, China). IVM, Cell Counting Kit-8 (CCK-8), and nigericin were obtained from MedChemExpress (Shanghai, China). Doxorubicin Hydrochloride Liposome Injection was supplied by Jiangsu Hengrui Pharmaceuticals Inc. (Jiangsu, China). IL-4 and granulocyte-macrophage colony-stimulating factor (GM-CSF) were bought from PeproTech (Rocky Hill, NJ, USA). The anti-mouse PD-L1 antibody (BE0246) was purchased from BioXCell. Alexa Fluor 488-conjugated calreticulin (CRT) antibody was obtained from Abcam (Cambridge, UK). DAPI (4′,6-diamidino-2-phenylindole) was supplied by KeyGEN BioTECH Corp. Ltd. (Jiangsu, China). The water used in experiments was purified through a Milli-Q system (Millipore, Burlington, MA, USA). All antibodies used for flow cytometry assays were commercially available, and their details are listed in Table S1. Primer sequences for reverse transcription-quantitative polymerase chain reaction (RT-qPCR) are detailed in Table S2. Other chemicals of analytical grade not mentioned above were also obtained from Sigma-Aldrich (St. Louis, MO, USA) and used as received without further purification unless specified otherwise.

### Synthesis of IMN

In this study, BMNs were synthesized via a facile one-step process using BSA as both the template and reductant, following a modified method previously reported by Chen et al. [20]. Specifically, a 4 mg ml^−1^ KMnO_4_ solution was prepared by dissolving 100 mg of KMnO_4_ in 25 ml of deionized water at room temperature. Subsequently, 25 ml of this freshly prepared KMnO_4_ solution was added dropwise to 25 ml (20 mg ml^−1^) of BSA solution under continuous stirring at 800 rpm at the same temperature, resulting in the formation of the BMN. The weight ratio of BSA to KMnO_4_ for the synthesis of BMN was 5:1. BMNs were purified by dialysis against deionized water for 24 h using an MD44 dialysis bag (Viskase, USA), with a 14-kDa molecular weight cutoff (MWCO) membrane, to eliminate unreacted components and residual impurities.

To prepare IMN, 20 mg of IVM was dissolved in 4 ml of anhydrous ethanol and then added dropwise to 80 ml of deionized water containing 80 mg of BMN, with constant stirring at 800 rpm at room temperature. After the addition, the mixture was allowed to react for 6 h under constant stirring at 800 rpm and room temperature. Following drug encapsulation, the IMNs were subjected to ultracentrifugation at 15,000*g* for 30 min to separate and remove any unencapsulated IVM. The supernatant containing the IVM-encapsulated nanoparticles was carefully collected. Then, the IMN suspension was reduced to a final volume of 10 ml by ultrafiltration with Amicon centrifugal filters (Millipore USA) using a 100-kDa MWCO membrane. This concentrate was then mixed with 3% (w/v) BSA and freeze-dried. The lyophilized IMN powder was stored at 4 °C for subsequent experiments.

More experimental section information can be found in the Supplementary Materials.

## Results and Discussion

### Preparation and characterization of IMN

Since IVM primarily binds to albumin in circulation [22], we explored albumin-based nanocomplexes for its delivery. Albumin nanomedicines have demonstrated high tumor-targeting efficiency and successful commercialization [23]. Therefore, we focused on designing albumin-based nanocomplexes to improve IVM delivery. Given the economic viability of BSA as an alternative to human serum albumin in numerous studies on nanomaterial-based antitumor immunity, our objective is to refine the therapeutic efficacy of IVM in cancer treatment through the use of BSA-based nanocomplexes. Compared to traditional MnO_2_ nanoparticle synthesis methods, protein-assisted reduction is a promising alternative. It enables rapid synthesis, eco-friendly conditions, and excellent biocompatibility. These unique properties make protein-assisted MnO_2_ nanoparticles particularly suitable for in-depth investigation as multifunctional nanotheranostic agents [24]. In this study, we capitalized on our previously developed MnO_2_-loaded BSA nanoparticles, referred to as BMN [20], as a carrier platform for IVM. Notably, BMN exhibited significantly improved IVM encapsulation efficiency, leading to the formation of IMNs. As detailed in Table S3, dynamic light scattering (DLS) measurements showed that the mean hydrodynamic diameters of BMN and IMN were 5.26 ± 1.25 nm and 78.34 ± 2.74 nm, respectively. In the preparation of BMN, MnO_2_ nanoparticles were generated by reducing KMnO_4_ (100 mg) with an excess of BSA (500 mg), in which the amino and sulfhydryl groups of BSA served as both reducing and stabilizing agents. This reaction yielded numerous small and BSA-stabilized MnO_2_ nanoparticles (BMN, ~5 nm). In contrast, the formation of IMN was driven by strong hydrophobic interactions between IVM and the BSA component within BMN, resulting in the assembly of larger nanoparticles (IMN, ~78 nm) [22]. A similar mechanism has been reported for albumin-bound paclitaxel (Abraxane, nab-paclitaxel), in which hydrophobic interactions between paclitaxel and albumin result in nanoparticles of ~130 nm [25,26]. The sub-100 nm size range of IMN is particularly advantageous, potentially facilitating passive targeting of drugs to tumors through the enhanced permeability and retention effect [27]. The zeta potentials of BMN and IMN were measured at −12.18 ± 1.27 mV and −14.83 ± 2.34 mV, respectively. The observed decrease in the zeta potential of IMN was likely attributed to the encapsulated IVM. The EE% and LC% of IVM in BMN were 82.24 ± 0.02% and 14.53 ± 0.04%, respectively. inductively coupled plasma optical emission spectrometry (ICP-OES) analysis showed that BMN contained 5.1 wt % Mn, while IMN contained 4.7 wt % Mn.

**Fig. 2.**
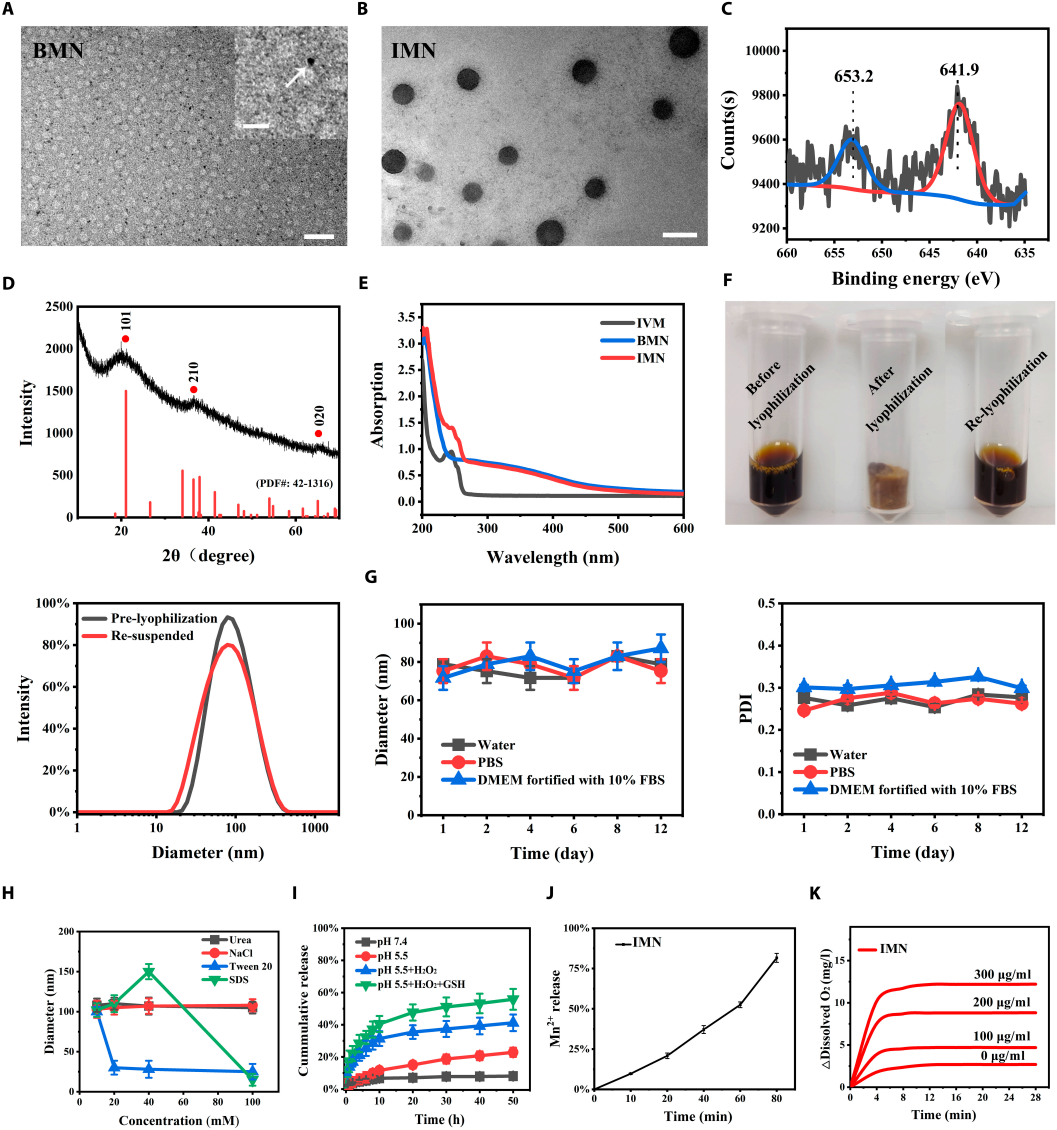
Characterization of nanoparticles. TEM images of (A) BMN (scale bars, 100 nm and 25 nm for the magnified image) and (B) IMN (scale bar, 100 nm). (C) XPS spectrum of the IMN. (D) XRD pattern of the IMN. (E) UV–vis absorption spectra of free IVM, BMN, and IMN. (F) Photographic representation of the aqueous solution of IMN, lyophilized form, and reconstituted state in a 0.9% NaCl solution post-lyophilization. Size distribution of the IMN prior to lyophilization and upon resuspension in a 0.9% NaCl solution. (G) Hydrodynamic diameter fluctuations and PDI variations of the IMN in various media over a 12-d period (*n* = 3). (H) Changes in the size of the IMN following exposure to differing concentrations of Tween 20, SDS, NaCl, and urea for 24 h (*n* = 3). (I) Cumulative release profile of IVM from the IMN under varying conditions (*n* = 3). (J) Accumulative Mn^2+^ release from IVM at pH 5.5 with GSH (5 mM) and/or H_2_O_2_ (100 μM), as a function of time (*n* = 3). (K) Oxygen generation from the IMN at different concentrations (*n* = 3).

Transmission electron microscopy (TEM) images (Fig. 2A and B) show that both BMN and IMN are uniformly dispersed and exhibit a spherical morphology. The average diameters are approximately 3.8 nm for BMN and 65.3 nm for IMN. Energy-dispersive spectroscopy (EDS) elemental mapping was performed to visualize the spatial distribution of Mn and O in both BMN and IMN. The results confirmed a uniform distribution of Mn and O within both nanomaterials (Fig. S1). X-ray photoelectron spectroscopy (XPS) was employed for further structural analysis, with representative spectra shown in Fig. 2C and Fig. S2 (full survey data). The spectrum displays 2 distinct peaks at binding energies of 653.2 and 641.9 eV, corresponding to the Mn(IV) 2p₁_/_₂ and Mn(IV) 2p₃_/_₂ spin-orbit split components, which are characteristic of MnO_2_. Figure 1D presents the x-ray diffraction (XRD) pattern of the IMN composite. The diffraction peaks at 2θ = 21.1° (101), 36.6° (210), and 65.1° (020) correspond to the ramsdellite–MnO_2_ phase (JCPDS No. 42-1316). Notably, the broadened peak profiles and reduced intensity of these reflections suggest diminished crystallinity of the ramsdellite–MnO_2_ component within the composite [28]. The synthesis of IMN was further confirmed by ultraviolet–visible (UV–vis) spectroscopy (Fig. 2E). The characteristic absorption peaks of IVM and BMN in the IMN spectra confirm the formation of complexes between IVM and BMN.

As depicted in Fig. 2F, after lyophilization with 3% (w/v) BSA as a cryoprotectant, the IMN formulations appeared as light brown solids with a spongy texture. Upon reconstitution with 0.9% sodium chloride (NaCl) solution, the nanoparticles rapidly dispersed and formed a uniform suspension. Importantly, the particle size of the IMN remained virtually unchanged before and after the lyophilization process. A basic stability study of the IMN was conducted for 12 d by monitoring changes in particle size and polydispersity index (PDI) across various media environments. Over the testing period, no significant variations in particle sizes or PDI were observed for the IMN suspended in water, phosphate-buffered saline (PBS), and Dulbecco’s modified Eagle’s medium (DMEM) containing 10% fetal bovine serum (FBS) at 37 °C (Fig. 2G). These data collectively underscore the excellent stability of IMN in various solvent systems. To delve deeper into the underlying formation mechanism of the IMN, we conducted experiments by introducing various concentrations of sodium dodecyl sulfate (SDS), NaCl, urea, and Tween 20 into the IMN solution. The findings depicted in Fig. 2H demonstrated that Tween 20 and SDS effectively disintegrated the IMN, suggesting a competitive hydrophobic interaction between these surfactants and the IMN. Conversely, urea, which is known for strong hydrogen bonding, and NaCl, which engages in ionic interactions, both failed to induce dissociation of the IMN. These results indicate that the primary interaction between IVM and BMN in IMN is predominantly hydrophobic rather than hydrogen bonding or ionic forces.

MnO_2_ is known to degrade in acidic TME, which is characterized by elevated levels of H_2_O_2_ and glutathione (GSH), a common feature of solid tumors. Considering this, we systematically explored the cumulative release profile of IVM from IMN across various aqueous solutions. As shown in Fig. 2I, IVM release from IMN was significantly slower at pH 7.4, with only 8.08 ± 0.02% released over 50 h. In contrast, under mildly acidic conditions (pH 5.5), there was a significant enhancement in the release rate, with 22.88 ± 0.03% of IVM being liberated within the same time interval. This accelerated release profile could be attributed to the increased decomposition rate of MnO_2_ in weakly acidic environments. Moreover, the presence of H_2_O_2_ led to a further accelerated release of IVM, reaching 41.20 ± 0.05% within 50 h. This observation suggests that H_2_O_2_ enhances MnO_2_ degradation under these weakly acidic conditions. Notably, the highest IVM release (55.82 ± 0.06%) occurred when both GSH and H_2_O_2_ were present at pH 5.5, indicating a synergistic effect of these 2 agents on MnO_2_ breakdown. In summary, our results collectively demonstrated that the IVM release behavior of IMN was tailored toward site-specific drug release within the TME, thereby reducing the potential for undesirable side effects on normal tissues.

The Mn^2+^ release under TME-mimicking conditions was also evaluated (Fig. 2J). Under pH 5.5 with H_2_O_2_ and GSH, Mn^2+^ was rapidly released from IMN, reaching 81.5% at 80 min. These results indicate that Mn^2+^ can be quickly released under conditions resembling the TME.

Given the overexpression of H_2_O_2_ within the concentration range of 10 to 100 μM in most types of solid tumors and considering the catalase-like activity of MnO_2_-based nanoparticles as reported by Chen et al. [20], we assessed the capacity of IMN to catalyze the decomposition of H_2_O_2_ into O_2_. Using a portable dissolved oxygen meter, we observed that IMN rapidly generated O_2_ in a dose-dependent manner, as depicted in Fig. 2K. In contrast, no increase in O_2_ concentration was observed in the absence of IMN. These findings suggest that IMN is capable of producing O_2_ utilizing H_2_O_2_ as a substrate, thereby potentially alleviating tumor hypoxia.

### Cytotoxicity of BMN and IMN

**Fig. 3.**
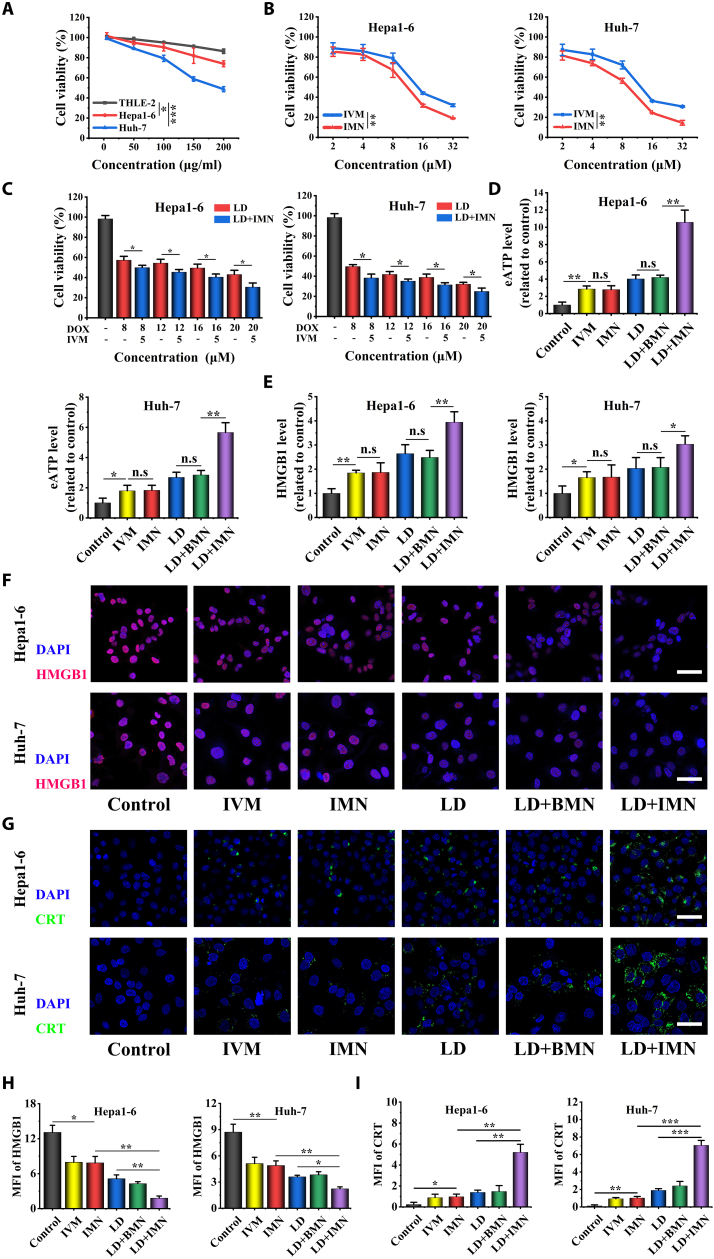
In vitro cytotoxicity and ICD evaluation of BMN and IMN. (A) Cell viability of Hepa1-6, Huh-7, and THLE-2 cells treated with the BMN at various concentrations (*n* = 6). (B) Viability of Hepa1-6 and Huh-7 cells treated with IVM and IMN at various concentrations (*n* = 6). (C) Cell viability of Hepa1-6 and Huh-7 cells treated with the combination of IMN and LD at various concentrations (*n* = 6). (D) eATP levels for Hepa1-6 cells and Huh-7 cells treated with saline, IVM, IMN, LD, LD + BMN, and LD + IMN (*n* = 3). (E) Extracellular HMGB1 levels for Hepa1-6 and Huh-7 cells treated with saline, IVM, IMN, LD, LD + BMN, and LD + IMN (*n* = 3). (F) Representative immunofluorescence images of HMGB1 in Hepa1-6 and Huh-7 cells under different treatments (red, HMGB1; blue, DAPI, scale bar, 50 μm). (G) Representative immunofluorescence images of CRT in Hepa1-6 and Huh-7 cells under different treatments (green, CRT; blue, DAPI; scale bar, 50 μm). (H) Quantification of mean fluorescence intensity (MFI) of HMGB1 determined using ImageJ. (I) Quantification of MFI of CRT determined using ImageJ.

It has been previously reported that the cytotoxic effects of MnO_2_ are attributed to the combined actions of Mn^2+^-mediated cytoplasmic DNA damage and GSH-depleting ferroptosis [29]. To evaluate the cytotoxicity of BMN against both hepatoma cells and healthy hepatocytes, we performed cytotoxicity assays using THLE-2, Hepa1-6 cells, and Huh-7 cells. As illustrated in Fig. 3A, a concentration-dependent cytotoxic response was observed for BMN. Notably, under the same BMN concentrations, THLE-2 cells showed higher viability than Hepa1-6 cells. Huh-7 cells exhibited the lowest survival rate among all tested cell lines. This suggests that BMN is less cytotoxic to normal cells than to hepatoma cells. This differential cytotoxicity might be attributed to the lower levels of H_2_O_2_ present in normal cells versus tumor cells and the inherent susceptibility of tumor cells to undergo ferroptosis [30,31].

The intracellular accumulation of IMN in different cell types was examined by confocal laser scanning microscopy (CLSM) (Fig. S3A). All tested cells showed time-dependent uptake of IMN-Cy5.5. Among them, Hepa1-6 cells exhibited the fastest and most efficient uptake, followed by Huh-7 cells, whereas THLE-2 cells showed the slowest uptake. Quantitative analysis of mean fluorescence intensity by flow cytometry confirmed these observations (Fig. S3B). Compared with the human normal liver epithelial cell line THLE-2, IMN-Cy5.5 was taken up more efficiently by the HCC cell lines Hepa1-6 and Huh-7.

Given the known preclinical anticancer potential of IVM, we investigated whether encapsulating IVM in IMN enhances its cytotoxicity against tumor cells [32]. As illustrated in Fig. 3B, both free IVM and IMN induced a pronounced, dose-responsive suppression of proliferation in Huh-7 and Hepa1-6 cell lines. Notably, IMN demonstrated significantly reduced IC_50_ (median inhibitory concentration) values of 11.02 and 8.25 μM for Hepa1-6 and Huh-7 cells, respectively, compared to the corresponding IC_50_ values of 16.36 and 13.63 μM for unencapsulated IVM. This reduction in IC_50_ values indicates a significant enhancement of IVM cytotoxicity upon encapsulation in IMN, confirming its role in augmenting IVM’s antitumor effect.

In clinical oncology, multidrug chemotherapy is common to enhance therapeutic outcomes, minimize side effects, and prevent drug resistance [33]. Prior research showed synergistic antitumor effects of IVM with doxorubicin and cisplatin [34,35]. Building on these results, we investigated whether IMN could boost the cytotoxicity of LD against Hepa1-6 and Huh-7 hepatocarcinoma cells. Notably, previous reports indicated that orally administered IVM achieved plasma concentrations reaching up to 5 μM without intolerable side effects [36]. Building on this evidence, we evaluated whether IMN, containing 5 μM IVM, could similarly enhance the cytotoxicity of LD against these liver cancer cells. Our experimental data, as shown in Fig. 3C, revealed an enhanced chemotherapeutic effect when IMN was combined with LD. This finding suggests a promising strategy for improving chemosensitivity in Hepa1-6 and Huh-7 cells while reducing toxicity associated with higher drug doses. These results highlight the critical role of IMN in enhancing LD effectiveness and suggest a pathway for developing novel combination therapies for HCC treatment. To further clarify the rationale underlying our combination strategy, we evaluated the synergistic effects of IMN and LD at various molar ratios in Hepa1-6 and Huh-7 cells. Among all tested ratios, the combination at an IMN:LD molar ratio of 1:10 exhibited the strongest synergistic effect, with combination index (CI) values of 0.41 in Hepa1-6 cells and 0.65 in Huh-7 cells at IC₅₀ (Fig. S4).

### In vitro evaluation of ICD

ICD is characterized by the extensive release of DAMPs, which enhance the immunogenicity of dying cancer cells and facilitate the recruitment and activation of antigen-presenting cells. Surface-exposed CRT, secreted eATP, and HMGB1 are paramount DAMPs associated with ICD [37,38]. Although doxorubicin effectively eradicates cancer cells, its ability to induce an immunogenic response is often overlooked due to a weak immune activation, which may contribute to tumor recurrence [19]. Motivated by previous findings highlighting the ability of IVM to trigger ICD [17,18], we systematically investigated whether coadministration of IMN alongside LD could enhance the induction of ICD. As shown in Fig. 3D, our findings show that LD and IMN combined treatment significantly increased eATP levels in Hepa1-6 and Huh-7 cells, exceeding those observed with LD or IMN monotherapy. Specifically, in Hepa1-6 and Huh-7 cells, LD + IMN treatment resulted in approximately 2.52-fold and 1.99-fold higher eATP levels than LD + BMN, highlighting the crucial role of IVM in stimulating eATP release. In contrast, the LD + BMN treatment group displayed eATP levels akin to those observed in the LD monotherapy group, indicating that BMN, at the administered dose, failed to elicit the significant production of eATP. Notably, both free IVM monotherapy and IMN monotherapy were capable of inducing substantial eATP generation, with no statistically significant differences between these 2 treatments. The release of eATP is an autophagy-dependent process, and inhibiting autophagy can significantly reduce eATP release during chemotherapy-induced ICD [7]. Previous studies have demonstrated the ability of IVM to induce autophagy via PAK1/Akt inhibition in breast cancer [18], and our study also found IVM-induced autophagy in Hepa1-6 and Huh-7 cells (Fig. S5). Consequently, IMN enhanced eATP release during LD-induced ICD by inducing both ICD and autophagy. Induction of autophagy is crucial for the antitumor immune response induced by chemotherapy. Unfortunately, autophagy is often suppressed during oncogenesis [7]. Prior research has shown that autophagy inducers can synergize with chemotherapy to enhance eATP release during ICD, significantly amplifying chemotherapy-induced antitumor immune activation [19]. These findings suggest that IMN, as a nanoscale autophagy inducer, has the potential to synergize with LD to enhance the antitumor immune response. Consistent with the eATP release results, combined treatment with LD and IMN markedly increased extracellular HMGB1 release in Hepa1-6 and Huh-7 cells compared with either LD or IMN alone. IMN monotherapy also induced a significant increase in extracellular HMGB1 release relative to the control group (Fig. 3E). Immunofluorescence analysis of nuclear HMGB1 further supported these findings: The combination of LD and IMN significantly reduced nuclear HMGB1 levels, and IMN alone also caused a marked reduction compared with the control group (Fig. 3F and H). As shown in Fig. 3G and I, CLSM imaging revealed varying levels of CRT exposure on Hepa1-6 and Huh-7 cell surfaces in all treatment groups, except for the control group. The LD + IMN treatment group exhibited significantly higher CRT exposure than the LD or IMN monotherapy groups, indicating a synergistic effect of IMN and LD on CRT exposure. Interestingly, the LD + BMN displayed CRT exposure levels similar to those in the LD group. These findings strongly suggest that IVM, rather than BMN, is the primary active component responsible for increased CRT exposure. Furthermore, IMN monotherapy alone induced significantly higher CRT exposure compared to the control group. In summary, our results demonstrate that IMN potentiated the ICD induced by LD, as evidenced by the elevated levels of ICD markers.

### Synergistic enhancement of DC maturation and NLRP3 inflammasome activation

**Fig. 4.**
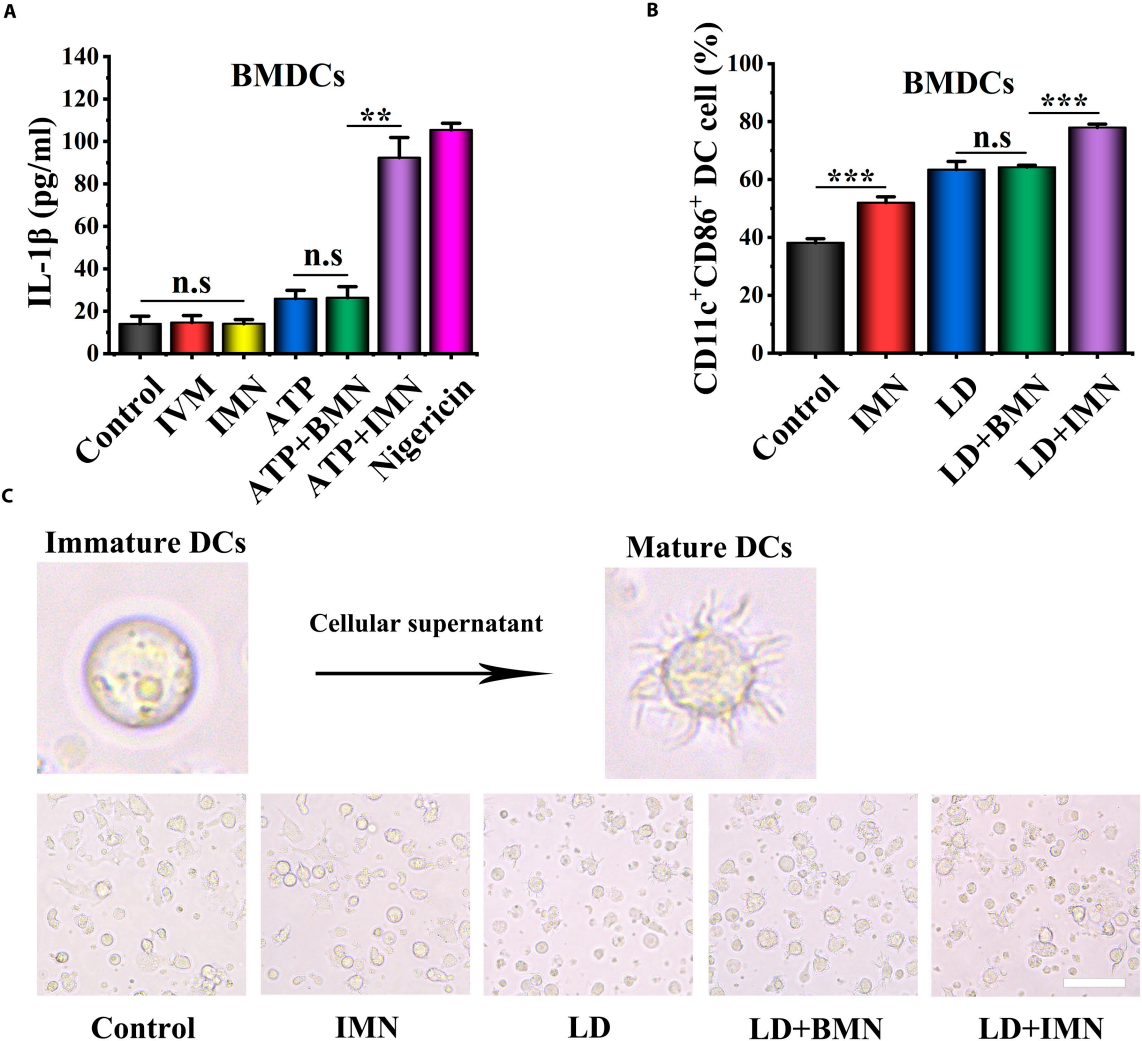
NLRP3 inflammasome activation in BMDCs by ATP and IMN cotreatment. (A) IL-1β release from BMDCs after various treatments (*n* = 3). (B) Flow cytometric analysis of BMDCs cocultured with supernatants from Hepa1-6 cells subjected to different interventions (*n* = 3). (C) Morphological changes in BMDCs induced by supernatants from Hepa1-6 cells treated with respective interventions (scale bar, 50 μm).

Among the P2X purinergic receptor family, P2X7R is the most challenging to activate. Its activation requires a sustained elevation of eATP concentration. Although some studies suggest potential compounds as positive allosteric modulators of P2X7 receptors, their clinical translation remains a lengthy process [39]. Overcoming this challenge involves screening approved drugs for new indications, an effective approach for expediting clinical translation. Extensive studies have revealed a synergistic interplay between P2X4R and P2X7R, demonstrating that the engagement of P2X4R potentiates P2X7R-mediated inflammasome activation and IL-1β secretion [40,41]. IVM has been characterized as a robust allosteric modulator of P2X4R [42], augmenting NLRP3 inflammasome stimulation by sensitizing P2X4R/P2X7R to eATP and enhancing IL-1β production by DCs [41,43]. Consequently, we investigated whether IVM-loaded IMN could sensitize P2X4R/P2X7R to eATP and enhance IL-1β production by DCs. The experimental data, as presented in Fig. 4A, clearly demonstrated that the combined therapy of ATP and IMN significantly boosted the secretion of IL-1β by bone marrow-derived dendritic cells (BMDCs) in comparison to ATP treatment alone. Notably, this combined regimen led to a 3.56-fold increase in IL-1β levels, underscoring the enhancing action of IMN on ATP-induced IL-1β release. In contrast, the administration of ATP + BMN elicited IL-1β secretion levels equivalent to those induced by ATP alone. This observation suggests that it was the IVM within IMN, rather than the BMN itself, that synergized effectively with ATP to stimulate IL-1β production. Furthermore, neither IMN nor free IVM significantly induced IL-1β release, suggesting that free IVM primarily modulates P2X7R sensitivity to eATP without directly activating the receptor. To delve deeper into the role of IMN, its effect on the DC2.4 cell line was examined, revealing a similar trend to that observed in BMDCs, yet with a lower amount of IL-1β release under identical conditions (Fig. S6A). Additionally, nigericin, a potent NLRP3 inflammasome agonist, was found to induce IL-1β release from BMDCs. In essence, this study confirmed that IMN could potentiate the activation of the NLRP3 inflammasome in DCs by sensitizing P2X7R to eATP, thereby leading to increased IL-1β secretion. This represents a significant advancement, as it establishes the role of IMN in sensitizing the ATP/P2X7R signaling axis in DCs, which plays a pivotal role in bridging innate and adaptive immunity.

To evaluate whether cell death induced by LD and BMN treatment could activate DCs, BMDCs were incubated with supernatants from Hepa1-6 cells treated with IMN and LD. Notably, the combined treatment of Hepa1-6 cells with IMN and LD induced the most pronounced BMDC maturation, as shown in Fig. 4B. For a comprehensive understanding of the gating strategy utilized in the flow cytometric analysis to ascertain the maturity of BMDCs, please consult Fig. S7. As shown in Fig. 4C, BMDCs exhibited significant morphological changes. Their membranes transitioned from a smooth surface (typical of inactive cells) to a tentacle-like pattern, indicating activation upon exposure to the supernatant from Hepa1-6 cells treated with IMN and LD. In contrast, this morphological change was not markedly observed for BMDCs exposed to supernatants from cells treated with IMN or LD alone. However, a similar morphological change was observed in BMDCs exposed to supernatants from cells treated with a combination of LD and BMN, suggesting a comparable effect on BMDC activation. Similar results were observed in DC2.4 cells (Fig. S6B and C). Collectively, these results indicate that IMN augments the immunogenicity of LD-induced ICD, thereby enhancing DC maturation.

### IMN alleviated hypoxia and suppressed adenosinergic axis in vitro

**Fig. 5.**
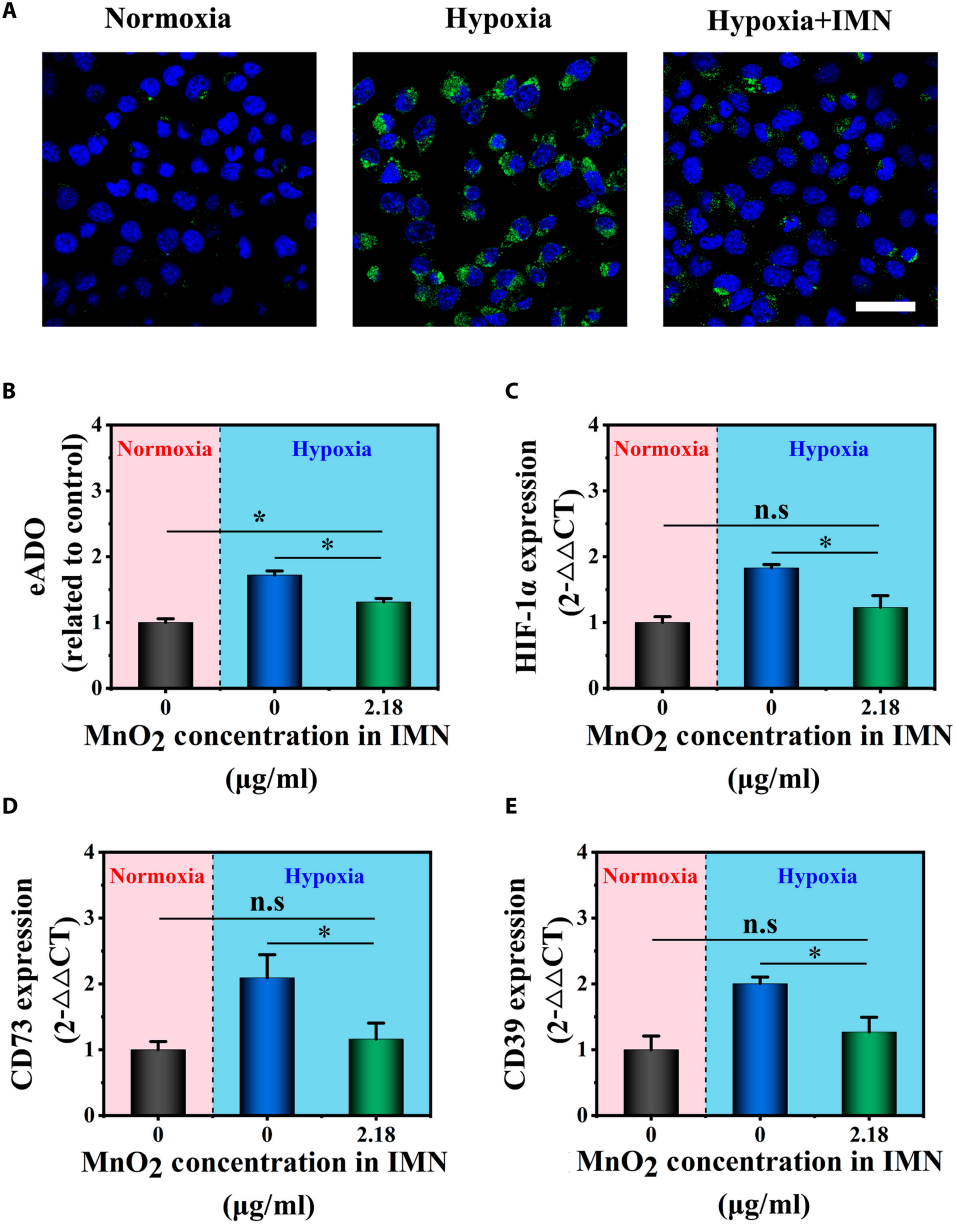
IMN alleviated hypoxia and modulated hypoxia-induced markers in Hepa1-6 cells. (A) Representative images of hypoxia detection in Hepa1-6 cells using Hypoxyprobe-1 staining under normoxic, hypoxic, and hypoxic + IMN conditions. Nuclei were stained blue with DAPI, while hypoxic cells appeared green (*n* = 3; scale bar, 50 μm). (B) Quantitative assessment of eADO levels in cell supernatants after 24 h of culture with or without IMN under normoxic or hypoxic conditions (*n* = 3). Expression levels of key molecular markers related to hypoxia-induced tumor aggression: (C) HIF-1α, (D) CD73, and (E) CD39 in Hepa1-6 cells after 24 h of incubation with varying concentrations of IMN under normoxic and hypoxic conditions (*n* = 3).

Preclinical evidence highlights the critical role of intratumoral hypoxia in stabilizing HIF-1α, a key regulator that enhances CD39 and CD73 transcription in the TME [10]. This up-regulation accelerates eATP degradation, hindering P2X7R activation and DC maturation, thereby compromising chemotherapy-induced ICD efficacy. MnO_2_ nanoarchitectures have garnered significant interest due to their ability to interact with elevated intracellular H_2_O_2_, continuously generating oxygen and alleviating tumor hypoxia in vivo [24]. Building on these findings and the experimental data in Fig. 1J, which demonstrated IMN’s ability to generate oxygen at physiologically relevant H_2_O_2_ concentrations in solid tumors, we investigated whether IMN could counteract the hypoxic–adenosinergic axis. To evaluate the impact of IMN on the hypoxic state within Hepa1-6 cells, we employed Hypoxyprobe-1 staining techniques. Hepa1-6 cells were cultured at 37 °C for 24 h under hypoxic conditions, with or without IMN treatment. As illustrated in Fig. 5A, untreated Hepa1-6 cells displayed a prominent green fluorescence indicative of Hypoxyprobe-1 binding, reflecting extensive hypoxia. In stark contrast, IMN-treated Hepa1-6 cells manifested a strikingly lower incidence of hypoxic cells, demonstrating the efficacy of IMN in alleviating hypoxia. Notably, under hypoxic conditions, IMN-treated Hepa1-6 cells showed no significant difference from those under normoxic conditions. This suggests that IMN effectively restored cellular oxygenation to near-normal levels. To further examine the effects of IMN on alleviating hypoxia, we measured eADO concentration in Hepa1-6 cell supernatants after 24 h of culture, with or without IMN treatment (Fig. 5B). In untreated Hepa1-6 cells under hypoxic conditions, eADO concentration was 1.72-fold higher than that under normoxic conditions. However, upon addition of IMN under hypoxic conditions, eADO concentration significantly decreased, nearly restoring it to normoxic levels. To assess the impact of oxygen environment modulation on the hypoxic phenotype of Hepa1-6 cells, we conducted a 24-h experiment where cells were exposed to either normoxic or hypoxic conditions, with or without IMN. Total mRNA was then extracted, and RT-qPCR analysis was performed to quantify the expression of key molecular markers strongly associated with hypoxia-induced tumor aggressiveness: HIF-1α, CD73, and CD39 (Fig. 5C to E). As expected, under hypoxic conditions, Hepa1-6 cells showed significantly higher expression levels of HIF-1α (+82.8%), CD73 (+109.3%), and CD39 (+100.3%) compared to normoxic conditions. Conversely, under hypoxic conditions, IMN treatment significantly reduced HIF-1α, CD73, and CD39 mRNA levels by 32.9%, 44.5%, and 36.7%, respectively, compared to the hypoxic control group. Similar results were obtained in Huh-7 cells (Fig. S8). In summary, our findings strongly support the notion that IMN effectively alleviates hypoxia, limits the generation of immunosuppressive adenosine, and suppresses hypoxia-induced immunosuppressive marker expression in cancer cells.

### Synergistic antitumor efficacy and safety profile of IMN combined with chemoimmunotherapy

**Fig. 6.**
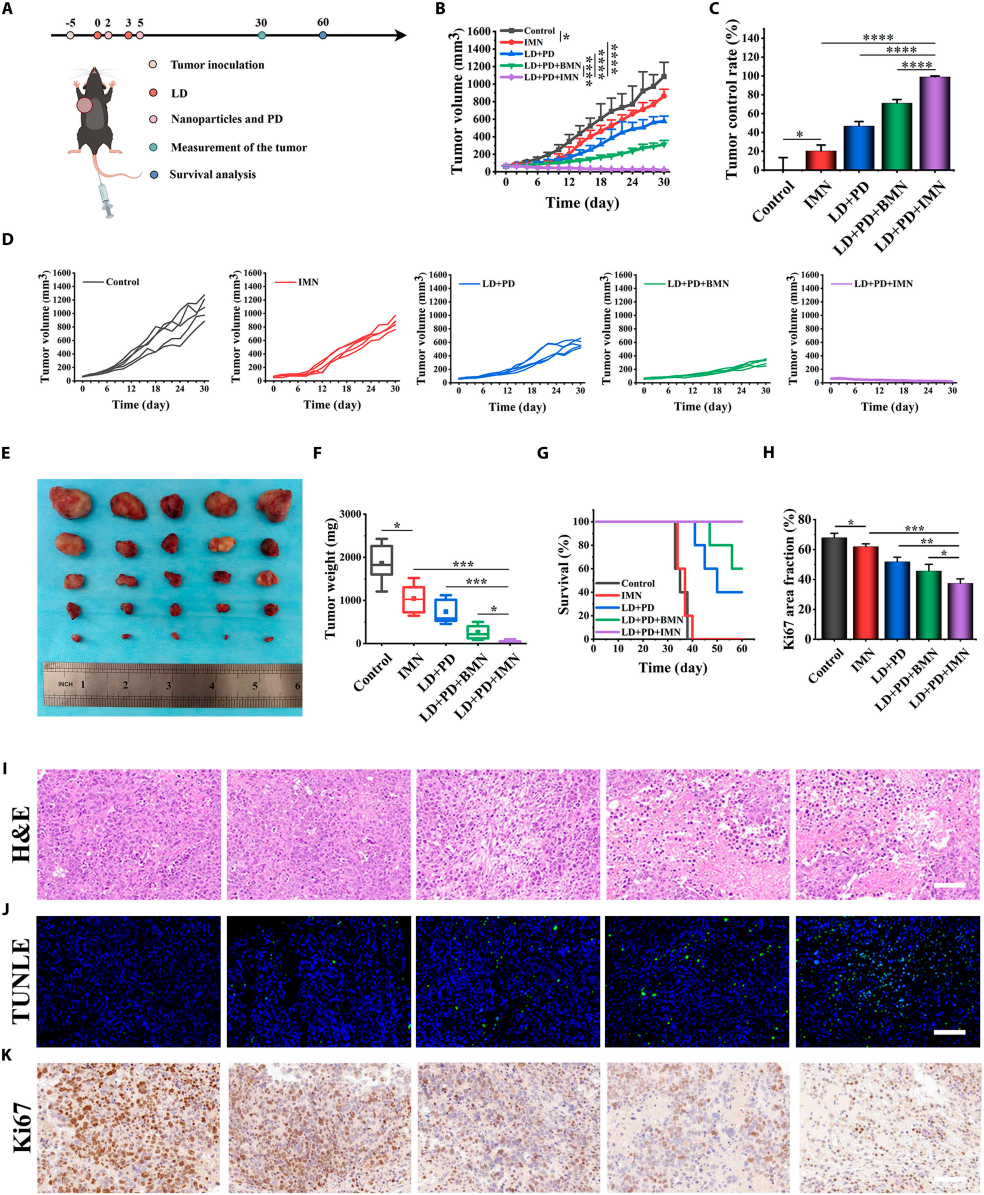
The antitumor effects of LD + PD + IMN in inhibiting primary tumors. (A) Schematic illustration of LD + PD + IMN-mediated antitumor experiment in a primary tumor model. (B) Tumor growth curves of the Hepa1-6 HCC-bearing C57BL/6J mouse model (*n* = 5). (C) Tumor control rate of the primary tumors (*n* = 5). (D) Individual tumor growth kinetics of the primary tumors were recorded every 2 d. (E) Images and weights of tumors obtained from Hepa1-6 HCC-bearing C57BL/6J mice on the 30th day after treatment. (F) Weights of tumors obtained from HCC-bearing C57BL/6J mice on the 30th day after treatment (*n* = 5). (G) Survival curves of each group. Representative (H) H&E, (I) TUNEL, and (J) Ki67 staining images of tumors collected from HCC-bearing C57BL/6J mice on the 30th day after treatment (scale bar, 100 μm). (K) Quantification of Ki67-positive area as percentage of total area (% area fraction) (*n* = 5). Positive cells or intensities were counted using ImageJ.

Building on the promising in vitro results demonstrating IMN’s efficacy in tumor therapy, we investigated its ability to enhance chemoimmunotherapy (LD + PD) in HCC animal models. Given the paramount importance of tumor-targeting efficiency in antitumor nanomedicine, we initially employed in vivo imaging techniques with Cy5.5-labeled IMN (IMN-Cy5.5), contrasting its performance with that of free BSA-Cy5.5. As shown in Fig. S9, our findings unveiled that IMN reached its peak tumor concentration 6 h after intravenous injection, approximately twice that of BSA. Furthermore, 24 h after administration, IMN exhibited significant accumulation in tumors, liver, and kidneys. This finding corroborates earlier results, which revealed intense Cy5.5-IMN fluorescence in the kidneys, likely due to renal clearance following nanoparticle breakdown [20]. This observation contrasted sharply with the absence of detectable fluorescence from BSA-Cy5.5 at the same time point. This underscores the superior targeting capabilities of Cy5.5-IMN, likely due to its optimized nanometric size, which enhances tumor delivery and retention. To validate the efficacy of IMN-augmented chemoimmunotherapy in retarding primary tumor growth, a murine Hepa1-6 subcutaneous tumor model was utilized. Upon tumors reaching a volume of roughly 100 mm^3^, mice were randomly assigned to 5 treatment groups and treated with saline, IMN, LD + PD, LD + PD + BMN, or LD + PD + IMN, as depicted in Fig. 6A, with dosages of 3 mg kg^−1^ doxorubicin (DOX), 5 mg kg^−1^ IVM, and 2.7 mg kg^−1^ MnO_2_. Tumor progression monitoring (Fig. 6B and D) clearly demonstrated that the LD + PD + IMN group almost completely suppressed tumor growth throughout the 30-d observation period. Tumor inhibition percentages were 20.43%, 46.88%, 71.19%, and an outstanding 98.94% for the IMN, LD + PD, LD + PD + BMN, and LD + PD + IMN groups, respectively (Fig. 6C). Although the LD + PD + BMN group also inhibited tumor growth, the effect was significantly less pronounced than that in the LD + PD + IMN group. These findings highlight the crucial role of IVM in enhancing the therapeutic potency of the LD + PD + IMN combination regimen. The therapeutic efficacy of this triple-combined therapy, consisting of LD + PD + IMN, markedly exceeded that of either the LD + PD dual therapy or IMN monotherapy, underscoring the synergy achieved by their concerted action. Notably, the IMN monotherapy group also showed greater tumor suppression than the saline group, suggesting that IMN has inherent antitumor activity. Throughout the observation period, saline-treated controls displayed rapid tumor progression. At the end of the experiment, tumor-bearing mice were euthanized, and their tumors were excised for weighing and photographic documentation (Fig. 6E and F). A 1-month post-treatment survival analysis showed that LD + PD + IMN significantly inhibited tumor growth, maintaining a 100% survival rate, whereas mice in other groups did not survive beyond days 33 to 60 (Fig. 6G). To elucidate the underlying mechanisms, we performed TUNEL (terminal deoxynucleotidyl transferase–mediated deoxyuridine triphosphate nick end labeling) fluorescence, hematoxylin and eosin (H&E), and Ki67 staining assays. Notably, tumors treated with LD + PD + IMN exhibited the strongest apoptotic signals. This was accompanied by minimal tumor cell proliferation, indicating enhanced apoptosis (Fig. 6H). These observations were consistent with histopathological evaluations using H&E staining, which further supported an elevated apoptotic response (Fig. 6I). Additionally, Ki67 staining assays echoed these findings, underscoring the significant apoptotic effect elicited by the LD + PD + IMN therapy (Fig. 6J and K). Monitoring of body weight revealed a consistent increase in the treatment groups, closely paralleling the trends observed in the control group (Fig. S10A). This finding provided initial evidence supporting the safety profile of the LD + PD + IMN therapy regimen. This safety profile was further supported by normal levels of hepatorenal function markers, including aspartate aminotransferase (AST), alanine aminotransferase (ALT), blood urea nitrogen (BUN), and serum creatinine (CR) (Fig. S10B). Hematological assessments concurred, showing no indications of infection or inflammation (Fig. S10C). Organ coefficient analysis and histopathological examinations (Fig. S10D to F) further confirmed that LD + PD + IMN did not cause significant adverse reactions. In summary, the integrated results from body weight monitoring, biochemical parameter assessments, histopathological evaluations, and hematological analyses collectively attested to the favorable safety profile of the LD + PD + IMN-based antitumor therapy regimen.

The therapeutic efficacy of LD + PD + IMN was further assessed in orthotopic Hepa1-6 HCC models (Fig. S11). In concordance with our observations from the subcutaneous tumor model, the hierarchy of therapeutic response regarding tumor volume diminution and growth arrest was as follows: control, IMN monotherapy, LD + PD, LD + PD + BMN, and, notably, LD + PD + IMN, which demonstrated the paramount inhibition of tumor progression. Remarkably, at the end of the observation period, the animal imaging system detected no tumors in any of the 5 mice receiving the LD + PD + IMN regimen, underscoring its exceptional antitumor potency.

### Immunological responses stimulated by LD + PD + IMN-mediated combined therapy

**Fig. 7.**
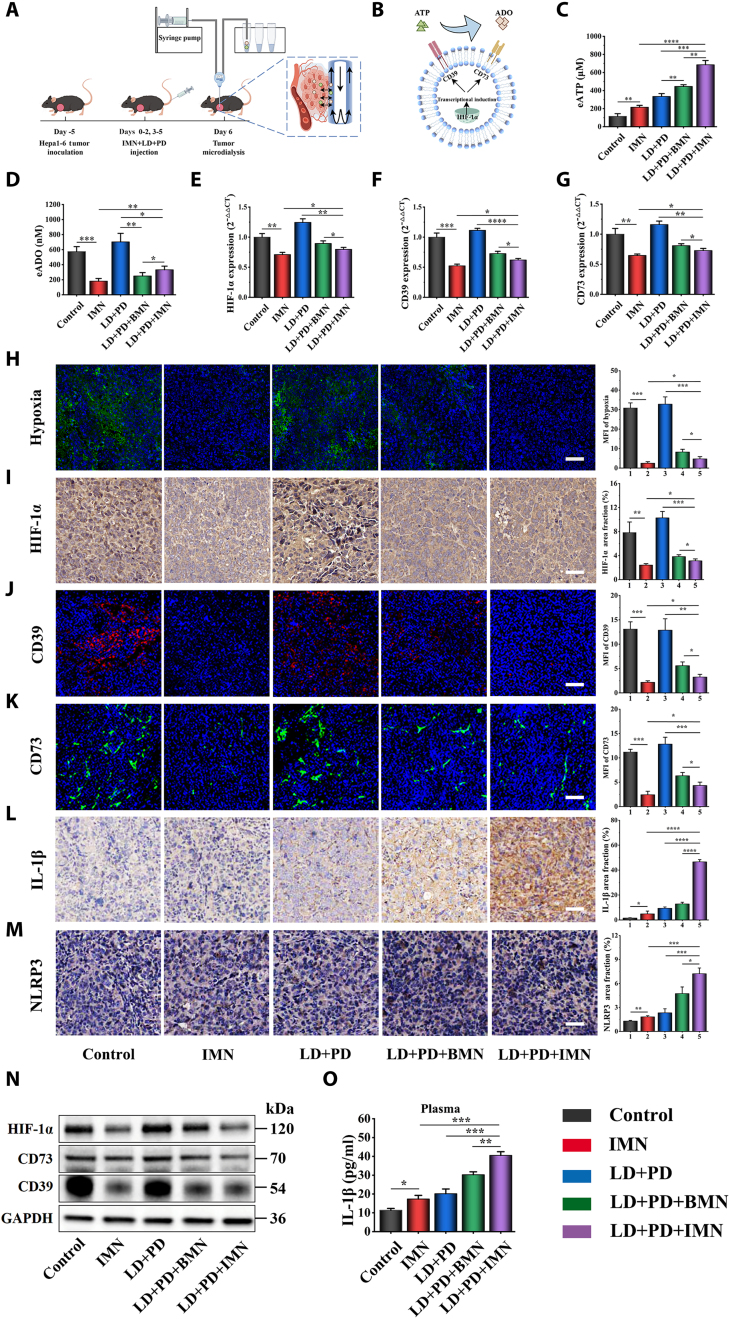
Levels of eATP and eADO, and expression of HIF-1α, CD39, CD73, IL-1β, and NLRP3 in tumors treated with the LD + PD + IMN combination therapy. (A) Schematic diagram depicting both the experimental design for in vivo quantification of eATP and eADO levels in the TME, and (B) the role of HIF-1α in up-regulating CD39 and CD73 expression for eATP conversion to eADO. Quantitative analysis of (C) eATP and (D) eADO levels in the TME, respectively (*n* = 3). RT-qPCR results revealing the relative mRNA expression of (E) HIF-1α, (F) CD39, and (G) CD73 in the TME (*n* = 3). (H) Representative immunofluorescence images showing the spatial distribution of pimonidazole (green), a hypoxia marker, in tumor tissue sections from different treatment groups. Nuclei were counterstained with DAPI (blue) (*n* = 3). Scale bar, 100 μm. The corresponding MFI was quantified using ImageJ. (I) Immunohistochemical staining images for HIF-1α expression in tumor tissues across various treatment groups (*n* = 3; scale bar, 50 μm). Quantification of HIF-1α-positive area as percentage of total area (% area fraction, *n* = 3). Positive cells or intensities were counted using lmageJ. Fluorescent multiplexed immunohistochemistry images of CD39 (J) and CD73 (K) expression in tumor sections from different treatment groups. Nuclei were counterstained with DAPI (blue) (*n* = 3; scale bar, 100 μm). The corresponding MFI was quantified using ImageJ. (L) Representative immunohistochemical images of IL-1β and (M) NLRP3 expression in tumor tissues from different treatment groups (*n* = 3; scale bar, 50 μm). The percentage of IL-1β- and NLRP3-positive area relative to total tissue area was quantified using ImageJ. (N) Representative Western blots showing the expression levels of HIF-1α, CD39, and CD73, normalized to glyceraldehyde-3-phosphate dehydrogenase (GAPDH) (*n* = 3). (O) Concentration of IL-1β in plasma among various treatment groups (*n* = 3).

Building on the promising in vivo results of the LD + PD + IMN regimen in orthotopic and subcutaneous tumor models, we investigated the immunological responses triggered by this combined therapy. To test this hypothesis, we used microdialysis to measure eATP and eADO levels in the TME after treatment. The treatment protocol and experimental setup for measuring eADO and eATP are outlined in Fig. 7A and B. C57BL/6J mice bearing Hepa1-6 HCC were randomly assigned to 5 treatment groups: saline (control), IMN-only, LD + PD, LD + PD + BMN, and LD + PD + IMN. eATP and eADO within the TME were quantified using microdialysis technology. A semipermeable probe with a double-layered membrane was inserted into the tumor on day 6. Continuous perfusion allowed eATP and eADO to diffuse from the TME into the dialysate across the membrane due to a concentration gradient. The dialysate was collected for subsequent quantification. The quantitative outcomes for eATP and eADO are delineated in Fig. 7C and D. The treatment with the LD + PD + IMN regimen elicited the highest eATP concentrations within the TME of tumor-bearing mice, surpassing all other therapeutic groups. This enhanced outcome was attributable to the multifaceted mechanisms of action inherent to the LD + PD + IMN regimen. In particular, this therapeutic approach effectively alleviated tumor hypoxia (Fig. 7H), which was corroborated by a marked diminution in the expression of HIF-1α, as quantitatively substantiated through RT-qPCR analysis (Fig. 7E) and qualitatively affirmed by immunohistochemical staining (Fig. 7I). The alleviation of hypoxia by the LD + PD + IMN regimen led to a decrease in CD39 and CD73 expression levels (Fig. 7F and G). This phenomenon was visually represented in Fig. 7J and K. The reduced expression of these ectonucleotidases curtailed the enzymatic conversion of eATP to eADO, thereby preserving higher eATP levels and enhancing the immunostimulatory potential of the TME. Consistently, Western blot analysis confirmed the reduced expression of HIF-1α, CD39, and CD73 (Fig. 7N and Fig. S12). Notably, the down-regulation of CD39 and CD73 curtailed the enzymatic conversion of eATP to eADO, thereby preserving higher eATP levels and enhancing the immunostimulatory potential of the TME. The observed reduction in CD39 and CD73 expression was consistent with previous research demonstrating that HIF-1α inhibition effectively suppresses these enzymes [11]. This supported our observation that the LD + PD + IMN regimen, by inhibiting HIF-1α, reduced CD39 and CD73 expression, thereby increasing eATP and decreasing eADO levels. Notably, despite IMN’s suppressive effect on CD39/CD73, eADO levels in the LD + PD + IMN group exceeded those in both the LD + PD + BMN and IMN monotherapy groups. This seemingly paradoxical finding could plausibly be attributed to the inherent capacity of IMN to induce ICD and to amplify ATP release triggered by LD. Consequently, the cumulative elevation of eATP, a direct result of the synergistic actions of IMN and LD, inadvertently fueled a heightened eADO production via sequential enzymatic breakdown. The administration of LD + PD + BMN notably augmented eATP concentrations in tumor tissues in comparison to the LD + PD regimen. This enhancement was ascribed to the capability of BMN to alleviate tumor hypoxia, thereby suppressing CD39 and CD73 expression and impeding the conversion of eATP to eADO. Consequently, the intervention of BMN not only escalated eATP levels but also concurrently decreased eADO within the TME. The LD + PD group displayed the most considerable accumulation of eADO, a consequence of the rapid enzymatic breakdown of a substantial quantity of LD-induced eATP into eADO, facilitated by the elevated expression of CD39 and CD73 in the TME. In contrast, IMN monotherapy, albeit inducing a less substantial rise in eATP concentrations compared to the LD + PD combination, uniquely demonstrated its efficacy through mechanisms involving hypoxia alleviation and suppression of CD39/CD73 expression. These actions effectively curtailed eADO accumulation, yielding the lowest quantifiable eADO levels. Ultimately, the LD + PD + IMN therapy had a dual effect on the TME, increasing eATP levels while reducing immunosuppressive eADO. This synergism effectively reshaped the TME into a more immunostimulatory setting, thereby inhibiting tumor progression.

Building on our initial findings, we assessed whether the elevated eATP levels induced by LD + PD + IMN could activate the NLRP3 inflammasome, leading to substantial IL-1β secretion. As visualized in Fig. 7L and M, tumors from Hepa1-6-bearing mice subjected to the LD + PD + IMN therapy displayed a pronounced accumulation of IL-1β and NLRP3, as evidenced by immunohistochemical analyses, relative to those treated with either LD + PD or IMN monotherapy. This robust expression of NLRP3, a pivotal constituent of the NLRP3 inflammasome, attested to superior activation of this inflammatory cascade by LD + PD + IMN. While the LD + PD and IMN-only therapies elicited some IL-1β release, the amounts were considerably lower. Conversely, negligible staining for IL-1β and NLRP3 was detected in tumors from the untreated control group. Notably, tumors from the LD + PD + BMN group exhibited diminished IL-1β and NLRP3 levels compared to the LD + PD + IMN group, implying the pivotal role of IVM in potentiating IL-1β and NLRP3 production. Serum IL-1β levels mirrored the patterns observed in tumor tissues, as shown in Fig. 7O, confirming the findings from tissue analysis. In summary, the synergistic LD + PD + IMN therapy stands out as the most efficacious in triggering the NLRP3 inflammasome pathway, leading to a substantial IL-1β secretion, outperforming other therapeutic regimens in terms of immune stimulation efficiency.

**Fig. 8.**
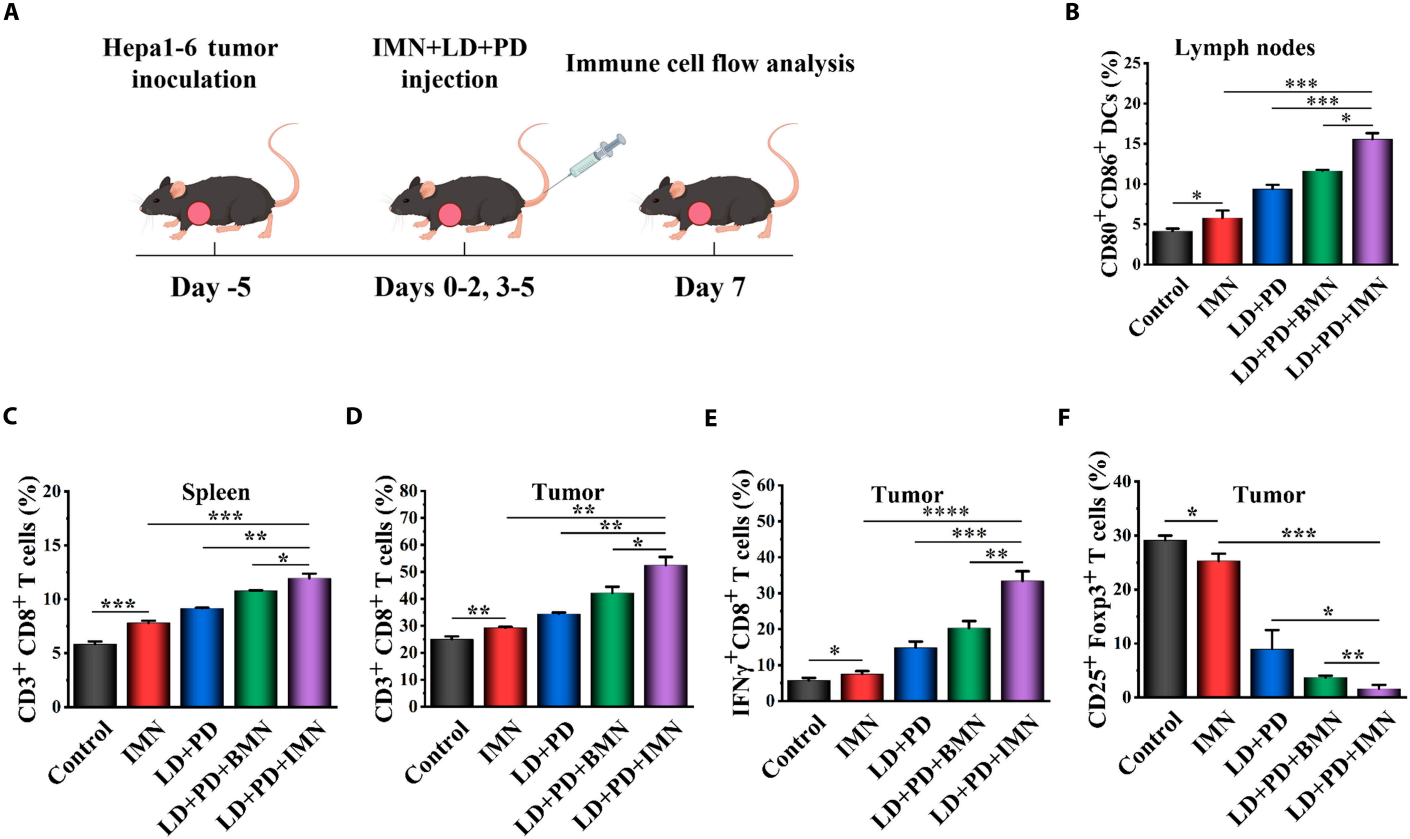
Characterization of immune cell populations. (A) Schematic representation of the establishment of the tumor-bearing mouse model and drug administration protocol for profiling immune cell populations. (B) Percentages of CD80^+^CD86^+^ DCs in tumor-draining lymph nodes (*n* = 3). (C) Percentages of CD3^+^CD8^+^ T cells in the spleen (*n* = 3). (D) Percentages of CD3^+^CD8^+^ T cells within CD3^+^ T cells in tumors (*n* = 3). (E) Percentages of IFNγ^+^CD8^+^ T cells within the CD3^+^ T lymphocyte population in tumors (*n* = 3). (F) Percentages of CD25^+^Foxp3^+^ Treg cells in tumors (*n* = 3).

IL-1β is a crucial inflammatory cytokine, with mature DCs being a significant source of it. IL-1β is then required for the adequate polarization of IFNγ-producing CD8^+^ T cells. To elucidate the mechanisms underlying the activation of DCs and IFNγ-expressing CD8^+^ T cells by the combined LD + PD + IMN therapy, we implemented diverse treatments in C57BL/6J mice bearing Hepa1-6 xenografts, adhering to the dosing regimen outlined in Fig. 8A. At 10 d post-intervention, tumor-draining lymph nodes, spleens, and tumors were harvested for comprehensive immunological evaluation. As shown in Fig. 8B, the population of mature DCs increased to 15.6% in mice receiving the LD + PD + IMN therapy, significantly surpassing the 11.6% and 9.3% observed in the LD + PD + BMN and LD + PD groups, respectively. These findings suggest that the LD + PD + IMN regimen demonstrated a superior capacity to promote DC activation compared to the LD + PD + BMN and LD + PD regimens. Moreover, IMN monotherapy also induced DC activation to some extent. Further analysis of CD8^+^ T cell activation in spleens and tumors (Fig. 8C and D) revealed that the LD + PD + IMN regimen elicited the highest proportions of CD3^+^ CD8^+^ T cells, reaching 11.9% in spleens and 52.4% in tumors, translating to 2.05-fold and 2.10-fold increments over saline controls, respectively. Additionally, Fig. 8E illustrated a substantial elevation in the frequency of IFNγ-producing CD8^+^ T cells across all therapeutic groups, with the LD + PD + IMN group demonstrating the most substantial elevation, a 5.86-fold increase over the saline control. This increase surpassed those observed in the LD + PD + BMN (3.56-fold), LD + PD (2.60-fold), and IMN-only (1.32-fold) groups, highlighting the efficacy of LD + PD + IMN in stimulating DCs and enhancing IFNγ-dependent CD8^+^ T cell responses essential for tumor elimination. The detailed gating strategies utilized for flow cytometric characterization of specific leukocyte subsets, namely, CD80^+^CD86^+^ DCs, CD3^+^CD8^+^ T cells in spleens and tumors, and IFNγ-secreting CD8^+^ T cells, were presented in Figs. S13 to S16.

The hypoxic nature of the TME is a well-established catalyst for enhancing regulatory T (Treg) cell up-regulation, a process that impairs the effectiveness of tumor-infiltrating lymphocytes, particularly cytotoxic CD8^+^ T cells, thereby fostering tumor progression. Given IMN’s ability to counteract hypoxia in tumor tissues, we performed flow cytometry to evaluate Treg populations under various treatment conditions. Figure 8F shows that the saline control group had a high percentage of CD25^+^Foxp3^+^ T cells (29.1%). The group treated with IMN alone also exhibited a reduction in Treg expression compared to the control group. The LD + PD group exhibited a significant reduction in Treg cell count, which fell to 8.9%, likely due to the off-target effects of LD [3]. Furthermore, the addition of BMN to LD and PD treatment resulted in an even lower Treg percentage (3.7%), indicating that BMN significantly mitigated hypoxia and suppressed Treg expression. Notably, the combination of LD, PD, and IMN reduced the Treg cell percentage to 1.6%, suggesting that IMN, possibly through the inhibitory action of IVM on Treg proliferation, further suppressed the Treg population. The gating strategy employed for flow cytometric analysis of CD25^+^Foxp3^+^ Treg cells was comprehensively detailed in Fig. S17.

In summary, our findings illustrate that IMN augments the release of eATP induced by LD therapy, enhancing the sensitivity of purinergic receptors P2X4R and P2X7R on DCs to eATP. IMN, by alleviating tumor hypoxia, suppresses the expression of CD39 and CD73 in the TME, thereby curtailing the conversion of eATP into the immunosuppressive eADO and concomitantly reducing the Treg population. This dual mechanism of IMN action elevates eATP concentrations in the TME and enhances NLRP3 inflammasome activation in DCs. This leads to increased production of the pro-inflammatory cytokine IL-1β and stimulates a broader repertoire of IFNγ-secreting, tumor antigen-specific CD8^+^ T lymphocytes. Consequently, this synergistic interplay enhances systemic and antigen-specific antitumor immune responses in vivo under PD therapy, highlighting the promising therapeutic potential of this combination regimen in cancer immunotherapy.

### The efficacy of LD + PD + IMN in suppressing distant tumor progression

**Fig. 9.**
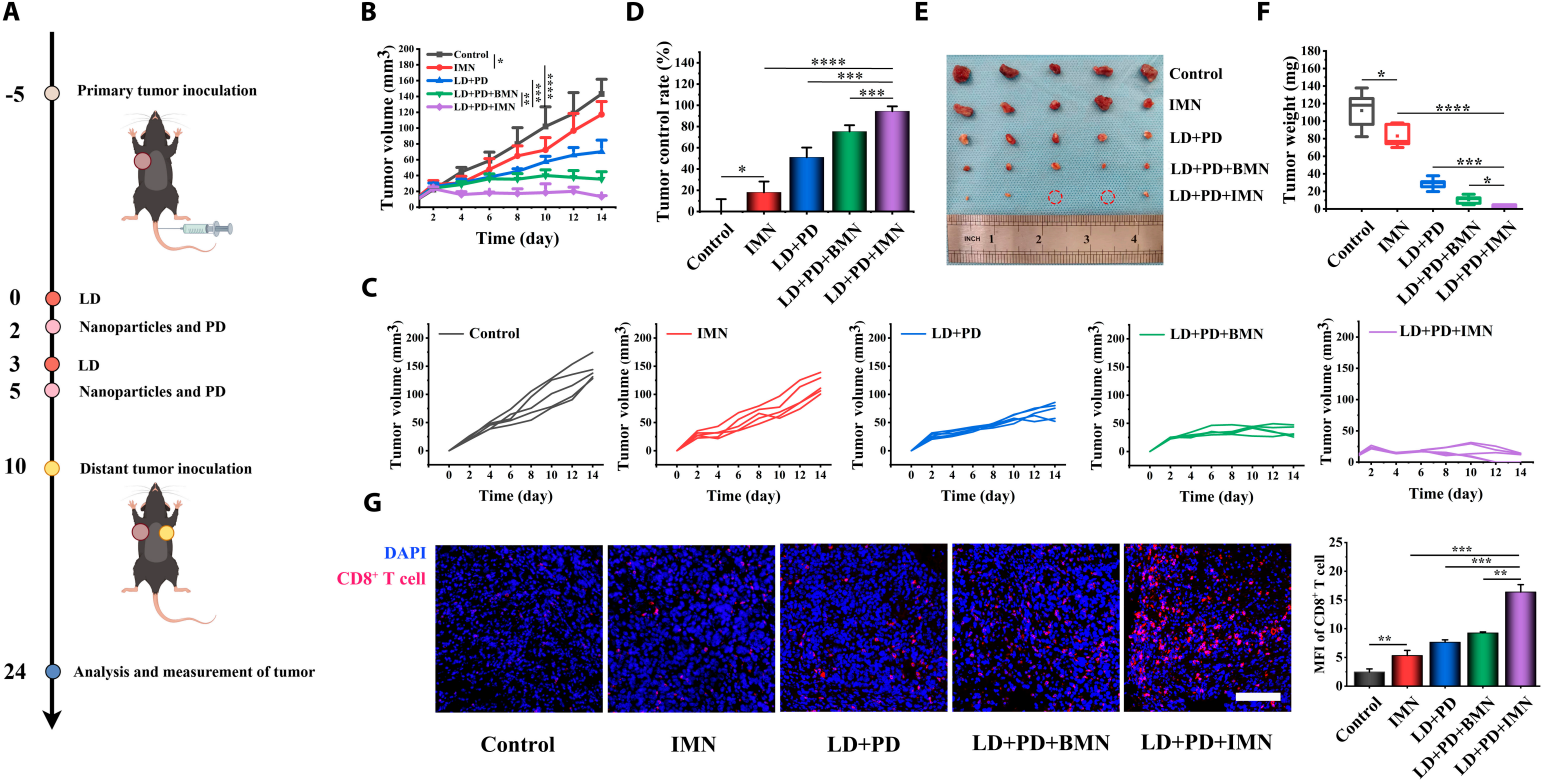
Distant tumor inhibition assay. (A) Schematic illustration of the experimental design with Hepa1-6-bearing C57BL/6J mice. (B) Distant tumor growth curves of the Hepa1-6-bearing C57BL/6J mouse model (*n* = 5). (C) Individual tumor growth kinetics of the distant tumors were recorded every 2 d (*n* = 5). (D) Tumor control rate of the distant tumors (*n* = 5). (E) Images and (F) weights of distant tumors obtained from Hepa1-6 HCC-bearing C57BL/6J mice (*n* = 5). (G) Representative immunofluorescence images of distant tumor tissues stained with DAPI (blue) and CD8^+^ T cells (red) in the different treatment groups (*n* = 3; scale bar, 100 μm). The corresponding MFI was quantified using ImageJ.

After evaluating the therapeutic efficacy and the robust antitumor immune responses induced by LD + PD + IMN, we investigated whether this regimen could inhibit distal tumor progression. A distal tumor model was established to assess whether the induced antitumor immunity could effectively inhibit the growth of untreated distant tumors after primary tumor intervention (Fig. 9A). As depicted in Fig. 9B and C, post-treatment monitoring of distant tumor dimensions revealed that the LD + PD + IMN group significantly reduced tumor volumes throughout the entire observation period, in stark contrast to the outcomes observed in the IMN monotherapy and LD + PD combination therapy groups. As shown in Fig. 9D, tumor inhibition rates were 18.07% for IMN, 51.01% for LD + PD, 75.30% for LD + PD + BMN, and a remarkable 94.40% for the LD + PD + IMN combination therapy. These observations implied that the synergistic action of IMN, in concert with LD and PD, engendered a more formidable immune response than individual therapies. Furthermore, the therapeutic regimen incorporating LD, PD, and IMN displayed a significantly greater capacity to inhibit the progression of distant tumors in comparison to the LD + PD + BMN group. This finding highlighted the essential role of IVM in enhancing antitumor immunity. In contrast, IMN monotherapy demonstrated limited efficacy in restraining tumor expansion, potentially due to insufficient stimulation of inherent antitumor immune responses. This observation was corroborated by the ex vivo image of excised tumors (depicted in Fig. 9E), which aligned with the terminal tumor weight data (depicted in Fig. 9F).

Since eATP activation of P2X7R triggers the NLRP3 inflammasome cascade, leading to IL-1β secretion—a key mediator of chemotherapy-induced antitumor immunity [6]—we investigated this pathway’s role in the immune response induced by LD + PD + IMN. Indeed, neutralization assays revealed that the blockade of IL-1β considerably undermined the therapeutic advantage (as shown in Fig. S18), highlighting the indispensable role of IL-1β. To investigate the mechanisms of enhanced tumor suppression, we conducted immunofluorescence analyses to quantify CD8^+^ T cell infiltration. Remarkably, the distant tumors treated with the LD + PD + IMN regimen exhibited a significantly greater infiltration of CD8^+^ T cells compared to those subjected to alternative treatments (illustrated in Fig. 9G). In summary, LD + PD + IMN therapy effectively suppressed distant tumor progression by inducing an abscopal immune response, highlighting its potential as a promising strategy in oncology.

## Conclusion

We demonstrated the pivotal role of IMN in potentiating chemoimmunotherapy by boosting NLRP3 inflammasome cascade in DCs—a pathway critical for chemotherapy-induced antitumor immunity. Our investigations revealed that IMN not only intrinsically induces ICD but also enhances LD-triggered eATP release by inducing autophagy and sensitizing P2X4R/P2X7R on DCs to eATP. Moreover, by alleviating tumor hypoxia, IMN reduced the expression of CD39 and CD73 on tumor cells, thereby decreasing eATP degradation and the immunosuppressive effects of eADO and Treg cells. These effects culminated in the robust activation of NLRP3 inflammasome signaling pathway in DCs, leading to substantial secretion of IL-1β, which was essential for the priming of IFNγ-producing, tumor antigen-specific CD8^+^ T cells. The combination of IMN with LD and PD led to near-complete inhibition of orthotopic and subcutaneous tumor growth. The marked reduction in distant tumor growth after treatment underscored the activation of a systemic immune response by IMN. In summary, our research provides substantial evidence supporting the integration of IMN into chemoimmunotherapy. This synergistic strategy holds significant promise for improving HCC treatment outcomes and may be applicable to diverse cancer types.

While this study presents promising findings, it acknowledges limitations that require further investigation. In our experimental design, LD, serving as the model drug, was administered systemically via tail vein injection, diverging from the local–regional interventional chemotherapy modalities such as TACE and HAIC commonly employed in the clinical management of HCC. Differences in administration routes (e.g., systemic versus locoregional) may affect therapeutic outcomes, necessitating comparative studies to evaluate their impact on efficacy. Moreover, the clinical dosage of IVM for tumor treatment must be finely adjusted to ensure therapeutic efficacy while maintaining acceptable side effects for cancer patients. Addressing these aspects will be crucial for translating our findings into clinical practice and maximizing IMN’s potential as a novel therapeutic modality.

## Ethical Approval

All animal procedures were meticulously performed in compliance with protocols sanctioned by the Ethics Committee of Fujian Medical University (IACUC FJMU 2023-Y-0100), adhering to the Guidelines for the Care and Use of Laboratory Animals and local regulations governing animal experimentation.

## Data Availability

Data will be made available on a reasonable request.

## Supplementary Materials

Supplementary 1 Experimental Section Figs. S1 to S18 Tables S1 to S3 References [44–47]
