# Supplementary material for: Purinergic Receptor Nanoimmunoamplifiers Potentiate Chemoimmunotherapy Efficacy in Hepatocellular Carcinoma
Source: Biomater Res. 2025 Nov 6;29:0278. doi: 10.34133/bmr.0278 (PMC12590479; doi:10.34133/bmr.0278)
Supplement: Supplementary 1 — Experimental Section Figs. S1 to S18 Tables S1 to S3 References [44–47] [file bmr.0278.f1.docx]

**Supporting Information**

**Experimental Section**

**Characterization of the nanoparticles**

The physicochemical properties of the synthesized nanoparticles were systematically characterized using various analytical techniques. The particle size, zeta potential, and polydispersity index (PDI) were measured using dynamic light scattering (DLS) with a Malvern Panalytical Zetasizer Nano series instrument (ZEN 3600, Malvern, UK). For morphological evaluation, transmission electron microscopy (TEM) was performed on a JEOL JEM-2100 microscope (JEOL, Tokyo, Japan). Energy-dispersive spectroscopy (EDS) elemental mapping was carried out using a field-emission transmission electron microscope (FETEM; Talos F200X, Thermo Scientific, USA). The manganese concentration in the nanoparticles was determined using inductively coupled plasma-optical emission spectrometry (ICP-OES) with an iCAP 7000 series spectrometer (Thermo Fisher Scientific, USA). X-ray diffraction (XRD) patterns were measured on a D/max-2500 X-ray diffractometer (Rigaku, Japan). To investigate the chemical state of MnO_2_, X-ray photoelectron spectroscopy (XPS) was performed using a Thermo Scientific K-Alpha XPS system (model Xi250, Thermo Fisher Scientific, USA). Lastly, the ultraviolet-visible (UV–vis) spectra of the nanoparticles were recorded using an UV-vis spectrophotometer (TU-1901 model, Puxi Instrument Co., Ltd., China).

For the assessment of encapsulation efficiency (EE%) and loading content (LC%), IVM was extracted from freeze-dried IMN (prepared in the "Synthesis of IMN" section) without the addition of BSA as a cryoprotectant. The concentration of extracted IVM was determined and calculated using a standard calibration curve, as measured by an UV-Vis spectrophotometer. The EE% and LC% for the IMN were calculated based on equations (1) and (2), respectively, with ma denoting the initial amount of IVM used for encapsulation, mf representing the actual amount of IVM quantified in the freeze-dried IMN, and mn indicating the weight of the resulting freeze-dried IMN. EE% was calculated using the following equation: EE% = mf/ma × 100% (1), LC% was determined as follows: LC% = mf/mn × 100% (2).

All measurements were performed using an ICP-OES instrument (iCAP 7000, Thermo Scientific, USA). The emission wavelength for manganese (Mn) was set at 257.61 nm with an integration time of 131 ms. The operating parameters were as follows: radio frequency power of 1275 W, auxiliary gas flow rate of 1.0 L min⁻¹, and nebulizer gas flow rate of 0.7 L min⁻¹. A MiraMist® parallel path micronebulizer was employed with a pump rate of 5 rpm (approximately 0.2 mL min⁻¹) and Tygon Orange/White pump tubing. Measurements were performed in radial plasma view. Mn content was calculated from the calibration curve (f(x) = 11848.2899x + 4.7083, R² = 0.9996). The background equivalent concentration (BEC) was 0.000 ppm, and the limit of detection (LoD) was 0.0007 ppm. Three independent samples of each nanoparticle (BMN and IMN) were dissolved in 10% aqueous HNO3 to prepare solutions at a concentration of 0.1 mg mL⁻¹. For each sample, 1 mL of solution was injected into the ICP-OES system to measure the Mn concentration. The Mn concentrations of the three BMN samples were 5.081, 5.122, and 5.103 ppm, with an average of 5.102 ppm. The Mn concentrations of the three IMN samples were 4.682, 4.725, and 4.706 ppm, with an average of 4.704 ppm. Based on these averages, the Mn content was calculated to be 5.1 wt% in BMN and 4.7 wt% in IMN.

The stability of the IMN was evaluated by monitoring changes in particle size and PDI over 12 days at 37°C. The investigation was conducted in three different media compositions: deionized water, phosphate-buffered saline (PBS, pH 7.4), and DMEM fortified with 10% fetal bovine serum (FBS).

In vitro release assays were carried out using a dialysis-based methodology. The process involved adding 1 mL of an IMN suspension (IVM dose: 500 μg mL^-1^) into a dialysis membrane bag with MWCO of 14 kDa, which was then immersed in 30 mL of PBS (pH 7.4 or 5.5) containing 0.15 g of sodium dodecyl sulfate (SDS) and stirred at 37°C at a constant speed of 100 rpm. To assess the effect of hydrogen peroxide (H_2_O_2_) and glutathione (GSH) on IVM release from IMN, additional control experiments were conducted using a protocol similar to that for pH-dependent IVM release (pH 7.4 or 5.5). H_2_O_2_ and GSH were introduced into the release medium at final concentrations of 100 µM and 5 mM, respectively. At predetermined time intervals, 1 mL aliquots of the release solution were collected and replaced with an equivalent volume of fresh release medium. The collected samples were subjected to freeze-drying before being reconstituted in 1 mL of methanol for IVM extraction. Subsequently, the solutions were centrifuged at 15000 g for 30 minutes at 4°C to separate supernatants. The concentration of released IVM in the supernatant was determined utilizing a standard calibration curve measured by UV-vis spectrophotometry.

The Mn²⁺ release profile of IMN was determined at 37 °C. IMN (40 mg) was dispersed in 4 mL of deionized water and loaded into a dialysis membrane bag (MWCO 3.5 kDa). The bag was immersed in 40 mL of PBS (10 mM, pH 5.5) containing H_2_O_2_ (100 µM) and GSH (5 mM). The system was stirred at 500 rpm at 37 °C. At predetermined time points (10, 20, 40, 60, and 80 min), 2 mL of the release medium was withdrawn and replaced with an equal volume of fresh medium. The collected samples were filtered through a 0.2 µm syringe filter. The concentration of released Mn²⁺ in each sample was quantified by ICP-AES using an iCAP 7000 series spectrometer (Thermo Fisher Scientific, USA).

O_2_ generation by IMN was quantified using a sealed chamber system with a dissolved oxygen meter (JPBJ-608). This system was equipped with microelectrode references and procured from INESA (Group) Co., Ltd, China. In the experimental protocol, 2 mL aliquots of IMN solutions at varying MnO_2_ doses, including 0 µg mL^-1^, 100 µg mL^-1^, 200 μg mL^-1^, and 300 μg mL^-1^, were dispersed in 10 mL of cell culture-grade PBS. Subsequently, H_2_O_2_ was introduced into the chamber to achieve a final concentration of 100 μM. Oxygen generation kinetics by the IMN were meticulously monitored at predefined time points.

**Cell culture**

The murine hepatoma cell line Hepa 1-6 (RRID: CVCL_0327, CTCC-400-0322); its luciferase-expressing derivative Hepa 1-6 Luc (CTCC-0436-Luc1), which was established by the stable transfection of the parental Hepa 1-6 cell line (RRID: CVCL_0327) with a luciferase reporter construct; the human hepatocellular carcinoma cell line Huh-7 (RRID: CVCL_0336, CTCC-003-0019); and the human normal liver epithelial cell line THLE-2 (RRID: CVCL_3803, CTCC-004-0030) were obtained from MeisenCTCC (Zhejiang Meisen Cell Technology Co., Ltd., Zhejiang, China). The official designations of the cell lines are Hepa 1-6, Hepa 1-6 Luc, Huh-7, and THLE-2. These cells were cultivated in DMEM supplemented with 10% FBS, 80 units mL^-1^ penicillin, and 80 µg mL^-1^ streptomycin at a standard culturing condition of 37°C under a humidified atmosphere containing 5% CO_2_. The THLE-2 cells were cultured in Bronchial Epithelial Cell Growth Medium enriched with 10% FBS, 5 ng mL^-1^ epidermal growth factor, 80 units mL^-1^ penicillin, and 80 µg mL^-1^ streptomycin, also incubated under the same standard conditions. The murine dendritic cell line DC2.4, generously gifted by Dr. Xiaojun Xia of Sun Yat-sen University, was cultivated in Roswell Park Memorial Institute 1640 medium (RPMI-1640) supplemented with 10% FBS and maintained at 37°C under a 5% CO_2_ atmosphere.

**Cytotoxicity assessment**

The cytotoxicity of BMN, free IVM, and IMN, as well as the combined treatment of LD and IMN, was evaluated using the CCK-8 assay. In detail, THLE-2, Huh-7, and Hepa1-6 cells were seeded in 96-well plates at a density of 1×10^4^ cells per well and allowed to adhere overnight. The medium was then replaced with fresh culture medium containing various concentrations of BMN, free IVM (prepared by diluting an IVM stock solution in dimethyl sulfoxide (DMSO) to achieve a final DMSO concentration less than 0.5% v/v in the cell culture media), or IMN, or a combination of LD (at a range of concentrations) with a fixed 5 μM concentration of IVM in IMN. Cells were incubated for an additional 48 hours at 37°C in a humidified atmosphere containing 5% CO_2_. After this period, the supernatants were carefully removed, and the wells were replenished with fresh medium containing 10% (v/v) CCK-8 reagent. The cells were incubated for 1 hour at 37°C in a 5% CO_2_ atmosphere. Cytotoxicity was quantified by measuring absorbance at wavelengths of 450 nm and 650 nm using the dual wavelength scanning functionality of a microplate reader (Model 680, Bio-Rad Laboratories, Inc., USA). The results represented the mean ± SD of six independent experiments.

**In vitro assessment of ICD**

To evaluate the induction of ICD, we monitored the release of eATP and high mobility group box 1 protein (HMGB-1) in both Hepa1-6 and Huh-7 hepatocellular carcinoma cell lines. Specifically, Hepa1-6 and Huh-7 cells were seeded at a density of 1×10^6^ cells per well in 6-well plates and incubated overnight to ensure proper adherence. Subsequently, the plates were then randomized into five treatment groups (n = 3/group): Group 1-Saline control, Group 2-Free IVM, Group 3-LD, Group 4-LD combined with BMN, and Group 5-LD combined with IMN. To ensure comparability, the final concentrations of DOX, IVM, and MnO_2_ were maintained at 5 μM, 5 μM, and 2.18 μg mL^-1^, respectively, across all treatment groups. In the groups designated for treatment comprising LD, the existing media were replaced with fresh culture medium supplemented with LD. Similarly, groups not receiving LD were replenished with fresh culture medium. Following a 48-hour incubation period, the remaining formulations were administered to the respective groups. For eATP quantification, cells were further incubated for 10 hours under standard culture conditions: in the dark at 37°C with 5% CO_2_. Following this incubation, conditioned supernatants were harvested through centrifugation at 4°C and 3000 g for 15 minutes to remove cellular debris and ensure sample purity. Immediately post-centrifugation, the concentration of eATP was determined using a commercial enhanced ATP assay kit according to the manufacturer's instructions (Cat. No. S0027, Beyotime). For the assessment of high mobility group box 1 (HMGB1) levels, a subsequent 24-hour incubation was carried out to capture late-stage ICD markers. A separate set of supernatants was collected following this extended incubation period, using identical procedures to those employed for eATP measurement. HMGB1 levels were measured using species-specific enzyme-linked immunosorbent assays (ELISA). The Mouse HMGB1 ELISA Kit (Catalog No. CSB-E08225m) and the Human HMGB1 ELISA Kit (Catalog No. CSB-E08223h) were used for Hepa1-6 and Huh-7 cells, respectively, following the manufacturers' protocols.

To enable direct visualization of HMGB1 release, immunofluorescence staining was performed on Hepa1-6 and Huh-7 hepatocellular carcinoma cell lines. Cells were seeded in 15-mm confocal dishes at a density of 1 × 10⁵ cells per dish and incubated overnight to allow attachment. Cell grouping and treatment protocols were identical to those used for the eATP assay. After treatment, cells were incubated for 24 h at 37 °C in the dark with 5 % CO_2_. Cells were then fixed with 4 % paraformaldehyde, rinsed with PBS, and incubated overnight at 4 °C with HMGB1 primary antibody (1:200; 82973-1-RR, Proteintech, China). Alexa Fluor 594-labeled secondary antibody (catalog no. A-21207, Thermo Fisher Scientific, USA; Ex/Em = 590/618 nm) was applied for 1 h at room temperature, followed by DAPI staining (Ex/Em = 405/466 nm) for 10 min. After extensive washing with PBS, samples were imaged using a confocal laser-scanning microscope (LSM980, Zeiss, Germany). Data represent three independent experiments.

To further investigate ICD in vitro, we monitored the surface exposure of calreticulin (CRT) in both Hepa1-6 and Huh-7 HCC cell lines. Cells were seeded onto laser confocal dishes (15 mm diameter) at a density of 1 × 10^5^ cells per dish and incubated overnight to ensure adequate attachment. The cell grouping and treatment protocols were identical to those used for the assessment of eATP and HMGB1. Following treatment administration, cells were incubated for 4 hours in the dark at 37°C with 5% CO_2_. Subsequently, fixation was achieved using a 4% paraformaldehyde solution, followed by rinsing with PBS. Cells were then exposed to an Alexa Fluor 488-conjugated CRT antibody (Ex/Em = 495/519 nm) for 1 hour at 4°C. Cells were stained with 4',6-diamidino-2-phenylindole (DAPI, Ex/Em = 405/466 nm) for 10 minutes to stain nuclei. After extensive washing with PBS, samples were analyzed under confocal laser scanning microscopy (CLSM; LSM980, Zeiss, Germany). Data are representative of three independent experiments.

**Ex vivo stimulation of BMDCs**

BMDCs derived from C57BL/6J mice were generated using a modified protocol based on the method described by Inaba et al. [44]. BMDCs were plated in a 48-well plate at a density of 5×10^5^ cells per well and incubated with Lipopolysaccharide (100 ng mL^-1^) overnight. The next day, the culture medium was replaced with 500 μL of fresh medium containing either free IVM, IMN, ATP, ATP combined with BMN (ATP+BMN), ATP combined with IMN (ATP+IMN), or nigericin to assess their effects on IL-1β release. To ensure uniformity, the final concentrations of ATP, IVM, and MnO_2_ were set at 1 mM, 5 μM, and 2.18 μg mL^-1^, respectively, across all treatment groups. Following a 3-hour incubation in the dark at 37°C with 5% CO_2_, supernatants were centrifuged at 2000 g for 20 minutes at 4°C, and 200 μL aliquots were stored at -80°C until assayed for cytokines via IL-1β mouse ELISA Kit (BMS6002, Invitrogen) according to the manufacturer's instructions.

To evaluate DC activation, Hepa1-6 cells were treated with free IVM, IMN, LD, LD+BMN, or LD+IMN for 48 hours. The final concentrations of DOX, IVM, and MnO_2_ were maintained at 5 μM, 5 μM, and 2.18 μg mL^-1^, respectively, in all groups. Supernatants from these treatments were then cocultured with BMDCs for 24 h. After the incubation, cells were imaged using an inverted microscope (Nikon TC-C-TC). BMDCs were then harvested, stained with FITC-CD11c and APC-CD86 antibodies, and analyzed by flow cytometry (BD FACSAria).

**In vitro characterization of the hypoxic-adenosinergic axis**

Cellular hypoxia in response to IMN was assessed using a Hypoxyprobe-1 plus kit (Hypoxyprobe, Burlington, MA, USA), following the manufacturer's recommendations. Huh-7 or Hepa1-6 cells were seeded at a density of 2×10^5^ cells per dish in laser confocal dishes (20 mm in diameter) and incubated overnight to allow adherence. Subsequently, the medium was replaced with either fresh culture medium (500 μL) or an equivalent volume containing IMN, with IVM and MnO_2_ concentrations set at 5 μM and 2.16 μg mL^-1^, respectively. Briefly, after a 24-hour incubation under normoxic or hypoxic conditions, cells were incubated for 2 hours with Hypoxyprobe-1(200 μM) and DAPI. The hypoxic cell growth conditions were established using an AnaeroPack™ MicroAero (Mitsubishi Gas Chemical, Tokyo, Japan) as reported previously [45]. Hypoxic cells were visualized using a Zeiss LSM 980 confocal microscope. Images were analyzed using Fiji software. The ratio of green (hypoxic) to blue (total) fluorescence was calculated to quantify hypoxia levels [46].

Adenosine concentrations were quantified using a fluorometric adenosine assay kit, following the manufacturer's protocol (ab211094, Abcam). Huh-7 or Hepa1-6 cells were seeded in a 6-well plate at a density of 5 × 10^5^ cells per well and incubated overnight to allow cell attachment. Then, cells were exposed to the same treatments under normoxic or hypoxic conditions. After 24 hours, supernatants were harvested and analyzed for adenosine content, with fluorescence intensity (Ex/Em = 535 nm/587 nm) measured using a SpectraMax M5 microplate reader (Molecular Devices, USA), normalized against an adenosine standard curve.

The gene expression of HIF-1α, CD39, and CD73 was assessed by RT-qPCR. Following a 24-hour incubation under normoxia or hypoxia, total RNA extraction was performed using an RNA isolation kit (TransGen Biotech ER501-01). cDNA synthesis was performed using a HiScript II 1st Strand cDNA Synthesis Kit (Vazyme, Nanjing, China) following the kit's instructions and using a Veriti 96 Well Thermal Cycler (ABI). RT-qPCR was performed using Taq Pro Universal SYBR qPCR Master Mix on the Applied Biosystems QuantStudio Plus real-time PCR system. Relative gene expression was calculated using 2^-ΔΔCT^ method, normalized to the housekeeping gene GAPDH.

**Animal experimentation**

Male C57BL/6J mice (3-5 weeks old, ~14 g) were procured from SiPeiFu Biotechnology Co., Ltd. (Beijing, China). All animal procedures were meticulously performed in compliance with protocols sanctioned by the Ethics Committee of Fujian Medical University (IACUC FJMU 2023-Y-0100), adhering to the Guidelines for the Care and Use of Laboratory Animals and local regulations governing animal experimentation.

**Tumor model establishment**

**Subcutaneous tumor model**

To establish a subcutaneous Hepa1-6 tumor model, male C57BL/6J mice aged between 3 and 5 weeks, with an average body weight of approximately 14 g, were employed in the experimental procedures. The tumor xenografts were established by subcutaneously injecting 3×10^6^ Hepa1-6 cells suspended in 100 μL PBS into the left flank region of each mouse. Tumor growth was monitored by measuring tumor volumes using the formula V = (W^2^×L)/2, where V represented the tumor volume, W was the width, and L denoted the length of the tumor.

**Orthotopic xenograft model**

An orthotopic Hepa1-6 cancer model was established by administering a 25 μL suspension of 5×10^5^ Hepa1-6-Luc cells mixed with 25 μL Ceturegel™ Matrix High Concentration Matrigel (Yeasen Biotechnology Co., Ltd., Shanghai, China; Catalog No. 40188ES08) into the liver lobes of mice. The presence of orthotopic tumors was confirmed by an IVIS Spectrum animal imaging system (PerkinElmer, USA) post-intraperitoneal luciferin injection, enabling visualization of bioluminescence from luciferase-positive Hepa1-6-Luc cells.

**Evaluation of in vivo anti-tumor efficacy**

Once the tumors reached an average volume of approximately 100 mm³ in the subcutaneous tumor model, mice were randomized into five treatment groups (n=10 per group): Group 1 (saline control), Group 2 (IMN), Group 3 (LD+PD), Group 4 (LD+PD+BMN), and Group 5: LD combined with PD and IMN(LD+PD+IMN). All treatments, excluding PD, were delivered intravenously at doses informed by pre-experimentation: DOX at 3 mg kg^-1^, IVM at 5 mg kg^-1^, and MnO_2_ at 2.7 mg kg^-1^ per mouse. PD was administered intraperitoneally at 5 mg kg^-1^. Treatment schedules were as follows: LD on days 0 and 3, with combinations involving IMN, BMN, and PD on days 2 and 5. Tumor volumes and mouse body weights were recorded bi-daily for 30 days. At the study endpoint, blood was collected via retro-orbital bleeding of all mice for ex vivo whole-blood analysis (RBC, WBC, PLT, HGB, and HCT). Serum was collected for biochemical analysis (AST, ALT, BUN, and Cr). Five mice from each group were then humanely euthanized, and their tumors were excised for weighing and further histological analysis including terminal deoxynucleotidyl transferase dUTP nick end labeling (TUNEL), hematoxylin and eosin (H&E) staining, and Ki67 immunohistochemistry. The remaining five mice per group were monitored for survival curve assessment.

To evaluate the in vivo anti-tumor efficacy in the orthotopic tumor model, mice with orthotopic tumors received the same treatments as those in the subcutaneous tumor model. Assessments of tumor volume in mice bearing orthotopic HCC were performed every 10 days using the IVIS Spectrum animal imaging system (PerkinElmer, USA).

**In vivo characterization of the hypoxic-adenosinergic axis**

To determine extracellular nucleotide levels, specifically eADO and eATP, an in vivo study was conducted using a murine hepa1-6 tumor model. C57BL/6J mice bearing Hepa1-6 HCC were randomly assigned to five therapeutic regimens (n=6 per group): saline (control), IMN alone, LD+PD, LD+PD+BMN, and LD+PD+IMN. Except for the intraperitoneally injected PD antibody at 5 mg kg^-1^, all interventions were administered intravenously at doses established from preliminary experiments: DOX at 3 mg kg^-1^, IVM at 5 mg kg^-1^, and MnO_2_ at 2.7 mg kg^-1^ per mouse. LD administration occurred on days 0 and 3, accompanied by the IMN and PD combination therapy on days 2 and 5.

Upon completion of the dosing regimen on day 6, three mice from each group were selected for microdialysis experiments. Microdialysis probes (CMA 12 Elite; 4 mm membrane, 20 kDa MWCO) were surgically implanted into tumors for sampling. The perfusate, composed of isotonic saline, 100 U mL^-1^ heparin, and 10 μM EHNA hydrochloride (adenosine deaminase inhibitor), circulated at 2 μL min^-1^ for 2.5 hours via a CMA 402 Syringe Pump. After probe stabilization, eADO levels were quantified using reverse-phase liquid chromatography-tandem mass spectrometry with a triple quadrupole instrument, as detailed in the Supplementary Materials. eATP levels were measured using the ATP Determination Kit according to the manufacturer's instructions.

C57BL/6J mice bearing Hepa1-6 hepatocellular carcinoma were randomly divided into five treatment groups (n = 3 per group): saline (control), IMN alone, LD + PD, LD + PD + BMN, and LD + PD + IMN. PD antibody was administered intraperitoneally at 5 mg kg⁻¹. All other agents were injected intravenously at doses determined from preliminary experiments: DOX 3 mg kg⁻¹, IVM 5 mg kg⁻¹, and MnO_2_ 2.7 mg kg⁻¹ per mouse. LD was given on days 0 and 3; IMN and PD combination therapy was administered on days 2 and 5. At the end of treatment, tumors were surgically excised and homogenized in ice-cold RIPA buffer (HY-K1001, MedChemExpress) supplemented with a protease inhibitor cocktail (HY-K0010, MedChemExpress). Tissue homogenization was performed at 4 °C using a tissue grinder (JXFSTPRP-24L, Shanghai Jingxin Industrial Development Co., Ltd., China). Homogenates were centrifuged at 12 000 g for 30 min at 4 °C, and the supernatant was collected. Protein concentrations were determined with a BCA Protein Assay Kit. Samples were mixed with loading buffer. For HIF-1α detection, samples were heated at 100 °C for 10 min; for CD39 and CD73, samples were heated at 50 °C for 10 min. All protein lysates were stored at -80 °C until use. Equal amounts of protein from each sample were loaded onto SDS-PAGE gels. Electrophoresis was carried out at 90 V until the bromophenol blue dye front reached the end of the gel. Proteins were then transferred onto polyvinylidene fluoride (PVDF) membranes at 350 mA for 40 min in an ice bath. Membranes were blocked in 5% non-fat dry milk for 2 h at room temperature and then incubated overnight at 4 °C with the following primary antibodies: anti-CD39 (Proteintech, #86327, 1:1000), anti-CD73 (Proteintech, #4434, 1:1000), anti-HIF-1α (CST, #ab36169, 1:1000), and anti-GAPDH (CST, #2118, 1:1000). After three washes with TBST, membranes were incubated for 1 h at room temperature with HRP-linked anti-rabbit IgG secondary antibody (CST, #7074, 1:3000). Following additional TBST washes, immunoreactive bands were visualized using SuperSignal™ West Pico PLUS Chemiluminescent Substrate (Thermo Scientific, #34580) and imaged with a ChemiDoc™ MP Imaging System (Bio-Rad Laboratories).

At the end of the treatment, blood was collected from the fundus of all mice to obtain plasma for analysis. Plasma IL-1β levels were quantified using an IL-1β mouse ELISA kit (BMS6002, Invitrogen) according to the manufacturer's instructions. A section of the excised tumor tissue was rapidly obtained for immunohistochemical analysis of CD73 and CD39, employing the tyramide signal amplification technique to increase assay sensitivity. Additionally, immunohistochemistry was performed to detect mouse IL-1β, HIF-1α, and components of the NLRP3 inflammasome. Primary antibodies (Servicebio, Wuhan, China) were utilized at a 1:200 dilution, specifically GB12115-100 for IL-1β, GB114936 for HIF-1α, and GB114987 for NLRP3. Additionally, total RNA extraction from tumor tissues facilitated gene expression analyses. Relative mRNA levels of HIF-1α, CD39, and CD73 were quantified via RT-qPCR, consistent with procedures outlined in the "In vitro characterization of the hypoxic-adenosinergic axis" Section.

After treatment completion, three mice from each group were selected to assess tumor hypoxia using the Hypoxyprobe™-1 Plus kit. Immediately after treatment ended, the mice received an intraperitoneal injection of Hypoxyprobe™-1 (pimonidazole HCl) at 60 mg kg^-1^ body weight. After one hour for probe accumulation, the mice were euthanized, and a segment of the excised tumor was quickly retrieved and sectioned. These tissue sections were subsequently stained with DAPI (dilution 1:500) to delineate cell nuclei and FITC-conjugated Mab1 (dilution 1:100) to identify hypoxic regions. Hypoxic regions within the TME were visualized using a Nikon Eclipse C1 confocal microscope.

**Immune cell phenotyping**

Once tumors reached approximately 100 mm³ in the Hepa1-6-induced model, tumor-bearing mice were randomized into five treatment groups (n = 3 per group): vehicle control, IMN monotherapy, LD+PD, LD+PD+BMN, and LD+PD+IMN. Intravenous administration was employed for all interventions, excluding PD, which was delivered intraperitoneally at a dosage of 5 mg kg^-1^. Dosages for DOX (3 mg kg^-1^), IVM (5 mg kg^-1^), and MnO_2_ NPs (2.7 mg kg^-1^ per mouse) were implemented as detailed in the "Evaluation of in vivo anti-tumor efficacy" section. The LD administrations occurred on days 0 and 3, with the IMN-PD combination therapy following on days 2 and 5. For comprehensive immune cell phenotyping, single-cell suspensions were prepared from tumor-draining lymph nodes (TDLNs), spleens, and tumors using cold PBS. These suspensions were fractionated for immunophenotyping of diverse leukocyte populations. Maturation status of DCs was assessed by staining of TDLN-derived cells with antibodies against CD11c, CD80, CD86, major histocompatibility complex class II (MHC-II), and MHC-I. CD8^+^ T lymphocyte populations were profiled from spleen extracts after staining with CD3, CD4, and CD8 markers. Cytotoxic T lymphocyte (CTL) characterization entailed the use of CD3, CD8, and IFNγ antibodies on tumor-infiltrating cell suspensions. Post-staining, samples underwent three rinses with PBS before being analyzed via a high-resolution multicolor BD LSR Fortessa X-20 Flow Cytometer.

**Assessing the systemic antitumor immune response in a bilateral tumor mouse model**

A bilateral tumor model was established to evaluate abscopal effects. Primary tumors were induced by subcutaneous injection of 3×10⁶ Hepa1-6 cells (in PBS) into the left flank of female C57BL/6J mice. Upon attaining an initial tumor volume of approximately 100 mm^3^ (designated as Day 0), mice were stratified randomly into five experimental groups (n = 5 per group): Vehicle control, IMN monotherapy, LD+PD, LD+PD+BMN, and LD+PD+IMN, in accordance with the predefined treatment regimen detailed in the "Evaluation of in vivo anti-tumor efficacy" section. Five days post-primary tumor intervention, distant tumors were initiated by subcutaneous injection of 2×10^6^ Hepa1-6 cells, suspended in PBS and admixed with an equivalent volume of Ceturegel™ Matrix High Concentration Matrigel (Catalog No. 40188ES08, Yeasen Biotechnology Co., Ltd., Shanghai, China) into the contralateral (right) flank. Subsequently, the growth of secondary tumors was monitored until the study ended. At the end of the study, tumors were surgically removed for immunofluorescence analysis targeting CD8^+^ T cells. Tumor specimens were rapidly frozen in liquid nitrogen and sectioned into 3 μm-thick slices using a Cryostat CM1950. (Leica Microsystems). These sections were stained with a primary antibody against mouse CD8 (GB15068, Servicebio, 1:300). After incubation, they were treated with a Cy3-labeled goat anti-rabbit secondary antibody (GB21303, Servicebio, 1:300) for fluorescence microscopy visualization.

**Statistical analysis**

Data are expressed as means ± standard deviation (SD). Differences between two groups were assessed for statistical significance using an unpaired, two-tailed Student's t-test. For multiple-group comparisons, one-way ANOVA with Tukey’s post hoc test (homogeneous variances) or Games-Howell test (heterogeneous variances) was applied. Results were considered statistically significant at p-values less than 0.05, with significance levels denoted as follows: *p < 0.05, **p < 0.01, ***p < 0.001, and ****p < 0.0001. Non-significant outcomes are indicated as n.s. Flow cytometry data were analyzed with FlowJo software (Version 10.8.1). The ImageJ software (National Institutes of Health; https://rsb.info.nih.gov/ij/) was used for the analysis of immunofluorescence data and immunohistochemistry data. Statistical analyses were conducted using OriginPro (Version 9.8.0.200). For the interpretation of in vivo bioluminescence assay data, Living Image software (Version 4.2.0.14335) was utilized.

**Supplementary experimental section**

**In vitro cellular uptake and flow cytometry**

Cells (15 × 10^4^) were seeded in 15-mm confocal dishes and incubated overnight to allow attachment. The medium was then replaced with fresh medium containing IMN-Cy5.5 (10 µM, based on Cy5.5) for 15 or 60 min. After three washes with PBS, cells were fixed with 4% paraformaldehyde for 30 min, stained with DAPI for 10 min, washed again, and imaged with a confocal laser-scanning microscope (LSM980, Zeiss, Germany). For flow cytometry, 1×10^5^ cells per well were seeded in 12-well plates and incubated overnight. The medium was replaced with fresh medium containing IMN-Cy5.5 (10 µM, based on Cy5.5) for 15 or 60 min. After three washes with PBS, cells were fixed with 4% paraformaldehyde for 30 min. Cellular uptake of IMN-Cy5.5 was then quantified by flow cytometry (CytoFLEX, Beckman Coulter) by measuring Cy5.5 fluorescence.

**Combination index analysis**

The combination index (CI) of IMN and LD was analyzed using CompuSyn software according to the Chou-Talalay method [47]. CI values were classified as synergistic (CI < 1), additive (CI = 1), or antagonistic (CI > 1). Seven molar ratios of IMN to LD (1:1, 1:2, 1:5, 1:10, 2:1, 5:1, and 10:1) were tested in Hepa1-6 and Huh-7 cells using the CCK-8 assay. Briefly, Hepa1-6 and Huh-7 cells were seeded in 96-well plates at a density of 5 × 10³ cells per well and allowed to adhere overnight. The medium was replaced with fresh culture medium containing different concentrations of IMN and LD at the indicated ratios. Cells were incubated for 48 h at 37 °C in a humidified atmosphere with 5% CO_2_. After incubation, the medium was removed and replaced with fresh medium containing 10% (v/v) CCK-8 reagent. Plates were incubated for 1 h at 37 °C, and absorbance was measured at 450 nm with a reference wavelength of 650 nm using a microplate reader (Model 680, Bio-Rad, USA). Data represent the mean ± SD of six independent experiments.

**Western blot analysis**

Adherent cells were gently washed twice with cold PBS and lysed in RIPA buffer (HY-K1001, MedChemExpress) containing a protease inhibitor cocktail (HY-K0010, MedChemExpress) for 30 minutes on ice. The lysates were then centrifuged at 14000 g for 30 minutes at 4°C to remove insoluble debris. The resulting supernatants were collected and total protein concentration was determined using a BCA protein assay kit (P0010S, Beyotime). Protein samples were subjected to sodium dodecyl sulfate-polyacrylamide gel electrophoresis and electrotransferred onto nitrocellulose membranes. The membranes were blocked with 5% skim milk in Tris-buffered saline with Tween 20 (TBST) for 1 hour at room temperature, followed by overnight incubation at 4°C with primary antibodies against LC3A/B (12741S, Cell Signaling Technology, diluted 1:1000) and β-actin (4970S, Cell Signaling Technology, diluted 1:1000). After thorough washing with TBST, membranes were incubated with HRP-conjugated secondary antibodies (HS101-01, TransGen Biotech Co., Ltd; 1:2000) for 1 hour at room temperature. The immunoreactive bands were visualized using SuperSignal West Pico PLUS Chemiluminescent Substrate (Thermo Scientific, 34580) and detected with a ChemiDoc MP Imaging System (Bio-Rad Laboratories).

**In vivo fluorescence imaging and biodistribution of BSA-Cy5.5 and IMN-Cy5.5 conjugates**

For the synthesis of IMN-Cy5.5, a solution comprising 100 μL of Sulfo-Cy5.5 NHS ester (R-FR-010, procured from Xi'an Rui xi Biological Technology Co., Ltd.), dissolved in dimethyl sulfoxide (DMSO) at a concentration of 5 mg/mL, was slowly added dropwise to a suspension of BMN in PBS (pH 7.4) containing 10 mg of BMN at 1 mg mL^-1^. The resulting mixture was gently agitated overnight to facilitate dye conjugation, yielding the fluorescently labeled BMN-Cy5.5. To remove unreacted Cy5.5 dye and residual DMSO, the BMN-Cy5.5 solution was subjected to extensive dialysis using a dialysis membrane with a MWCO of 14 kDa for a duration of 24 hours. Upon completion of dialysis, the BMN-Cy5.5 were freeze-dried to produce a blue-brown amorphous powder. IMN-Cy5.5 were synthesized by leveraging BMN-Cy5.5 as the initial substrate, meticulously following the established protocol outlined in the "Synthesis of IMN" section. BSA-Cy5.5 nanoparticles were synthesized using BSA as the primary material, following a protocol analogous to that employed for the preparation of BMN-Cy5.5. The exact quantity of Cy5.5 dye integrated into both BSA-Cy5.5 NPs and IMN-Cy5.5 NPs was determined using a Cary Eclipse fluorescence spectrophotometer (Agilent Technologies G9800a, Malaysia), with excitation and emission wavelengths set at 680 nm and 720 nm, respectively.

C57BL/6J mice bearing tumors were randomly assigned into two experimental groups. Each mouse in the respective group was intravenously administered via the tail vein with a single dose of either 100 μL BM-Cy5.5 NPs or IMNS-Cy5.5 NPs dissolved in saline solution at a cy5.5 concentration of 2.5 mg/kg body weight. Subsequently, the biodistribution and tumor accumulation of the nanoparticles were assessed by whole-body imaging using an IVIS Lumina X5 Imaging System（PerkinElmer, US) equipped with specific fluorescent filter sets calibrated for excitation/emission wavelengths of 680 nm/720 nm. Imaging was performed at predefined time points post-administration: 1, 2, 4, 6, 8, 12 and 24 hours. Upon reaching the 24-hour mark following injection, the mice were euthanized, and their major organs, including hearts, livers, spleens, lungs, kidneys, and tumors, were harvested. The collected organs were then imaged under identical conditions using the living body imaging system to evaluate the distribution and potential organ-specific accumulation of the Cy5.5-labeled NPs.

**Measurement of adenosine by mass spectrometry**

Adenosine was purchased from MedChemExpress (HY-B0228, Shanghai, China). All reference standards were preserved at -20°C. Methanol, acetonitrile and analytical-grade formic acid suitable for LC-MS-MS, were sourced from Merck Inc. (Darmstadt, Germany). Quantification of adenosine was performed using selective reaction monitoring on an UPLC-QTOF-MS system (AB QTRAP 5500, USA). High-performance liquid chromatography (HPLC) was conducted using a Thermo Fisher Hypersil GOLD™ C18 column (150 × 2.1 mm, 3 µm). The mobile phase was composed of two buffers: Buffer A, consisting of 0.1% formic acid in water, and Buffer B, a solution of 0.1% formic acid in acetonitrile. The flow rate of the mobile phase was set at 400 μL/min, and the column temperature was maintained at 40°C.The injection volume was 1 µL. The total run time for the analysis method was 2 min. Adenosine were determined by triple-quadrupole tandem mass spectrometry with an electrospray ionization interface running in positive ionization mode. The selected reaction-monitoring transitions of m/z 268.1 [M+H]^+^→m/z 268.1 for Adenosine were used with dwell time of 100 ms. Cone voltage was set at 25 V for adenosine. The Analyst® software (Version 1.7.3) was used to control the UPLC–MS/MS system. Data were collected and processed using MultiQuant™ software (Version 3.3.1.43). This method was validated in microdialysate solution with a daily working range of 25-2550 pM.

**Impact of IL-1β neutralization on systemic antitumor immune efficacy in a bilateral HCC model**

To investigate the importance of NLRP3 inflammasome activation for the abscopal therapeutic effect on tumors distant from the primary site, we assessed the systemic antitumor immune efficacy of LD+PD+IMN in the presence or absence of neutralizing IL-1β with anti-IL-1β antibodies (BE0246, BioXCell). Specifically, 3×10^6^ Hepa1-6 cells suspended in PBS were subcutaneously implanted into the left flank of female C57BL/6J mice, creating the primary tumor. Once the primary tumor reached an initial volume of approximately 100 mm³ (designated as Day 0), mice were randomly divided into three groups (n = 5 per group): Saline control, LD+PD+IMN, and LD+PD+IMN+anti-IL-1β. The dosages were in accordance with the predefined treatment regimen detailed in the "Evaluation of In Vivo Anti-Tumor Efficacy" section. Anti-IL-1β was administered intraperitoneally at 1 mg kg^-1^. The treatment schedules were as follows: LD and anti-IL-1β on days 0 and 3, with IMN and PD administered on days 2 and 5. Five days post-primary tumor intervention, distant tumors were initiated by subcutaneously injecting 2×10^6^ Hepa1-6 cells suspended in PBS and mixed with an equal volume of Ceturegel™ Matrix High Concentration Matrigel (Catalog No. 40188ES08, Yeasen Biotechnology Co., Ltd., Shanghai, China) into the contralateral (right) flank. The development of these secondary tumors was closely monitored until the end of the study.


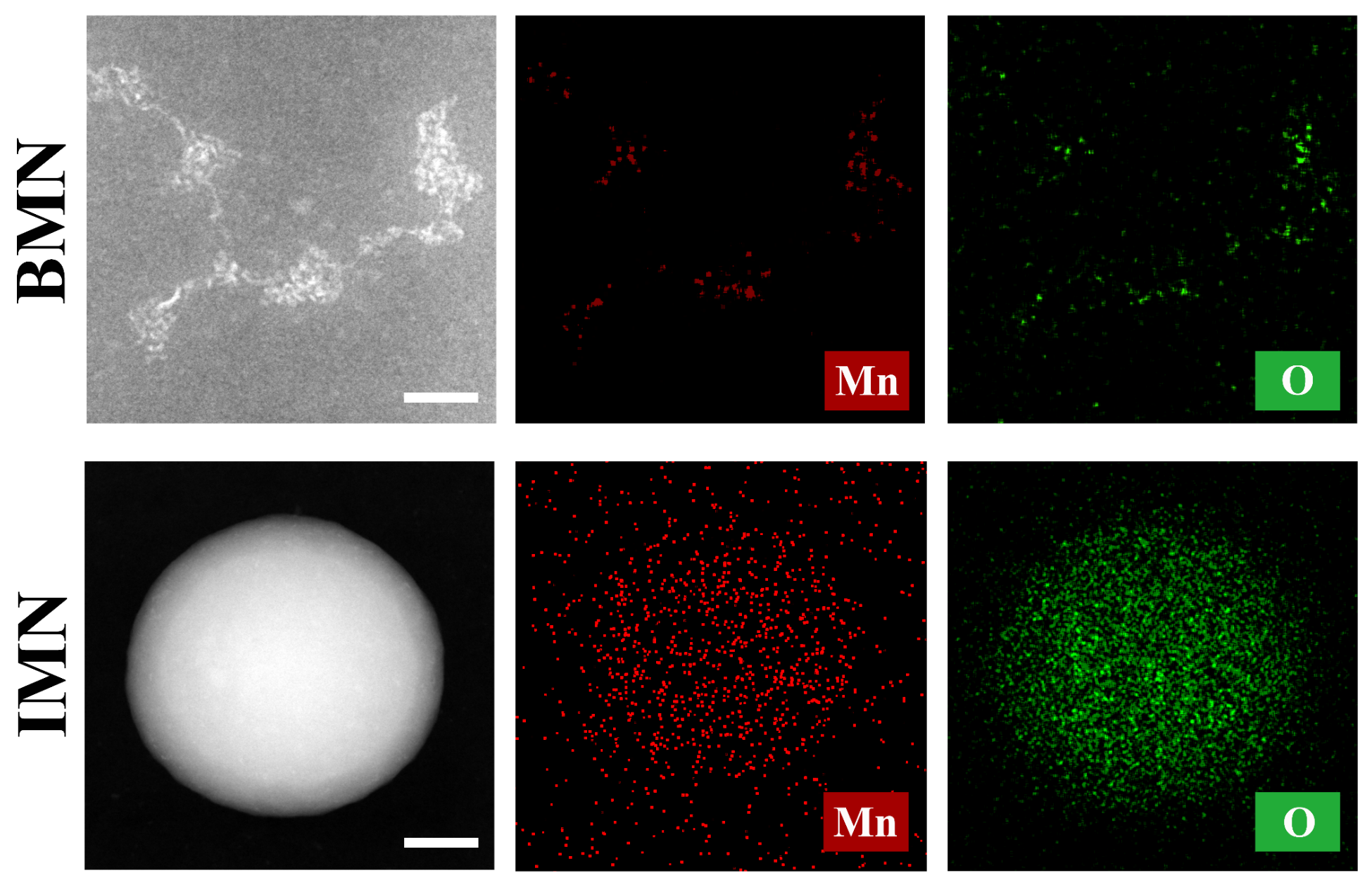


**Figure S1.** EDS elemental mapping of BMN and IMN, with scale bars set at 5 nm and 20 nm, respectively. The signals of manganese and oxygen are shown in red and green, respectively.


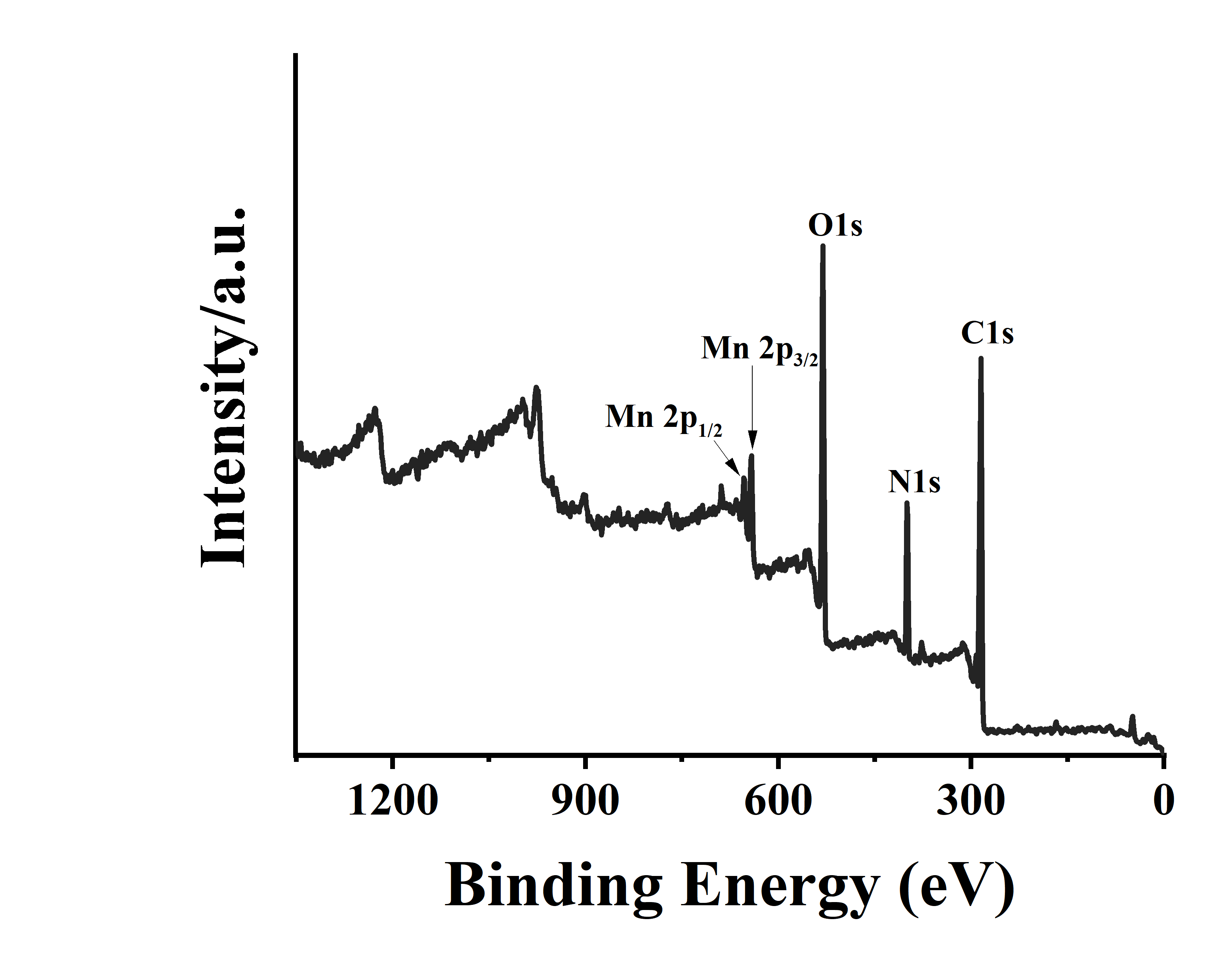


**Figure S2.** Full-survey XPS spectra of IMN.


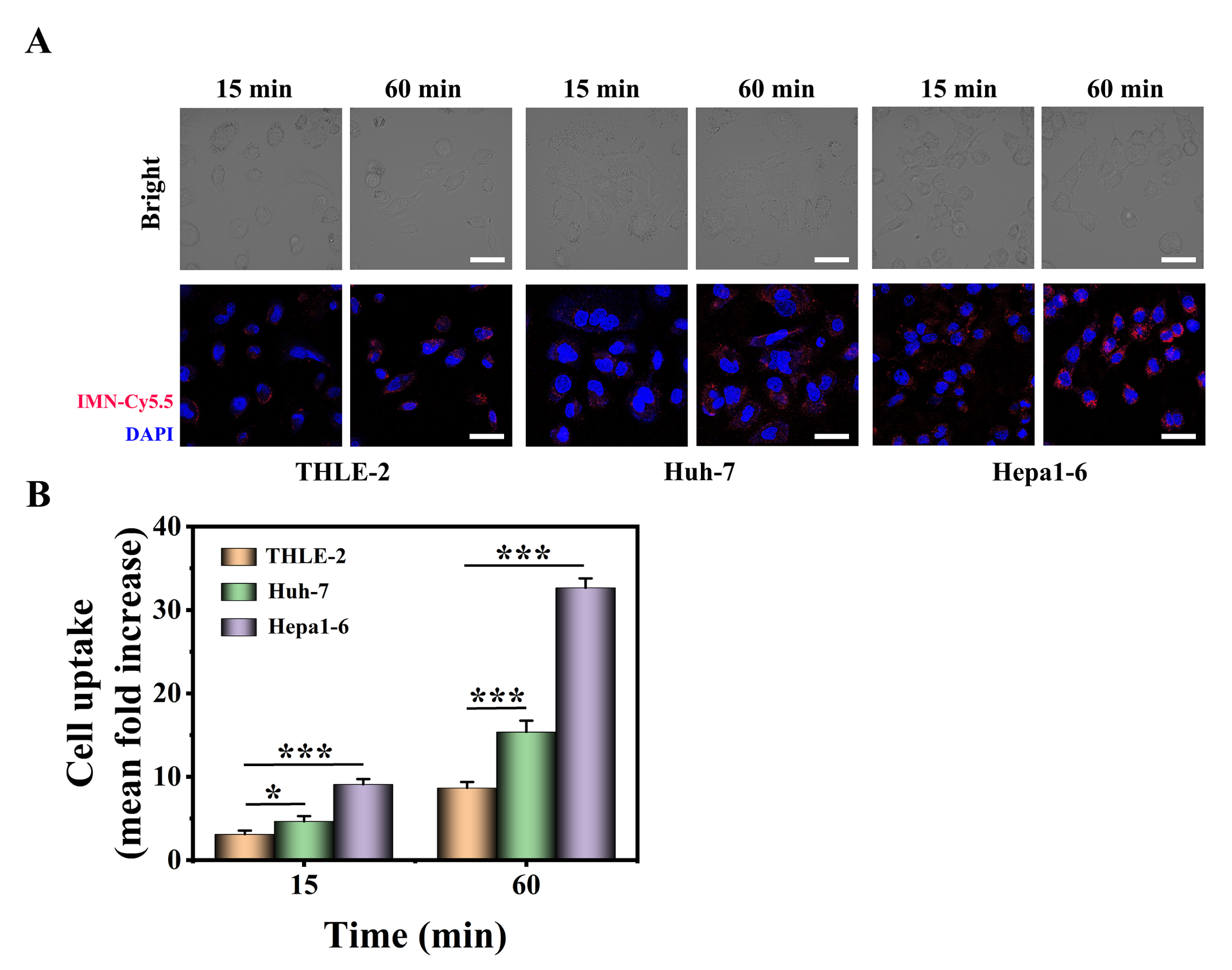


**Figure S3.** (A) Confocal images of Hepa1-6, THLE-2, and Huh-7 cells incubated with IMN-Cy5.5 for different time points (Scale bar = 20 µm). (B) Quantitative flow cytometry analysis of cellular uptake. Mean fluorescence values were normalized to the total fluorescence intensity of the corresponding untreated cells (without Cy5.5-IMN).


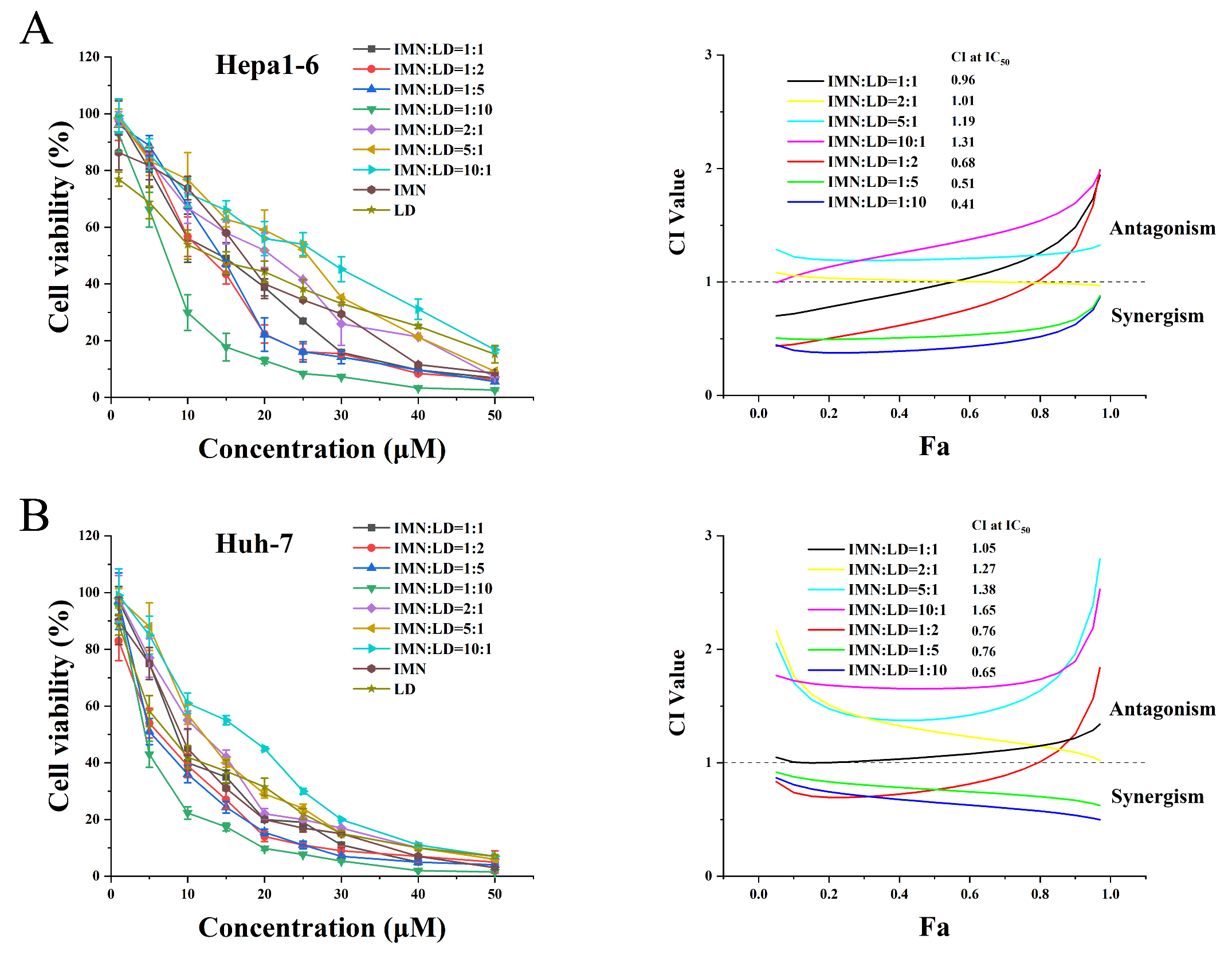


Figure S4. Cytotoxicity and CI values of IMN and LD in (A) Hepa1-6 and (B) Huh-7 cells. Various molar ratios of IMN and LD were tested using the CCK-8 assay. CI values were calculated with CompuSyn software according to the Chou-Talalay method. CI < 1 indicates synergism, CI = 1 indicates additivity, and CI > 1 indicates antagonism.


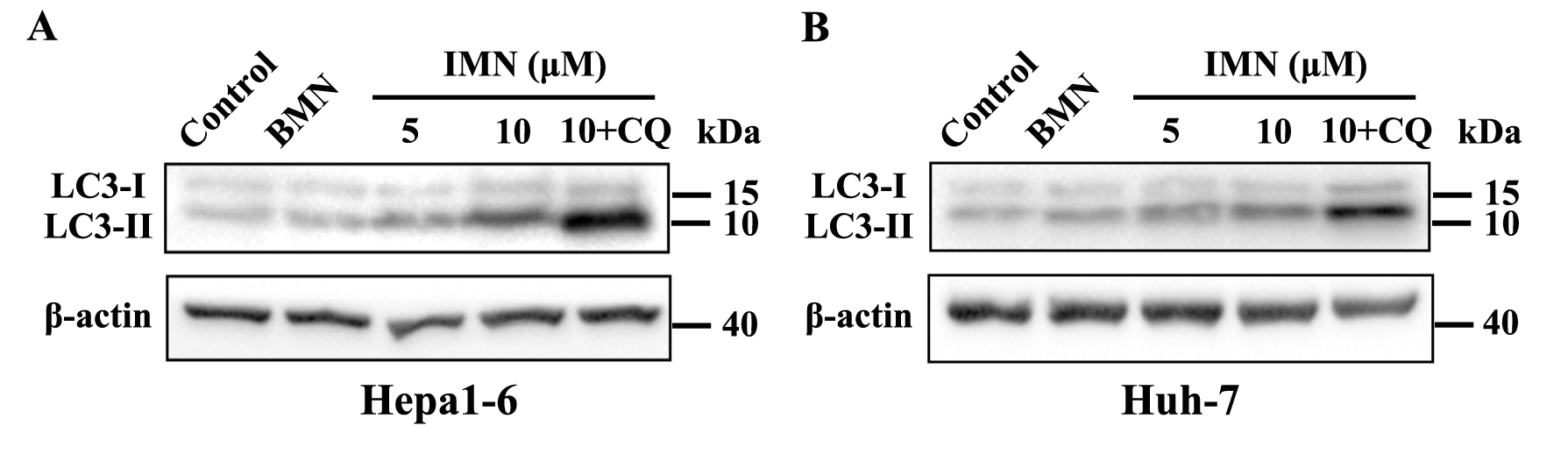


**Figure S5.** IMN-induced autophagy in Hepa1-6 and Huh-7 cells. Western blot analysis of LC3-I and LC3-II protein levels in (A) Hepa1-6 and (B) Huh-7 cells treated with increasing doses of BMN or IMN alone or IMN in combination with chloroquine (CQ, an autophagy inhibitor; 10 μM) for 24 hours. β-actin was used as a loading control.


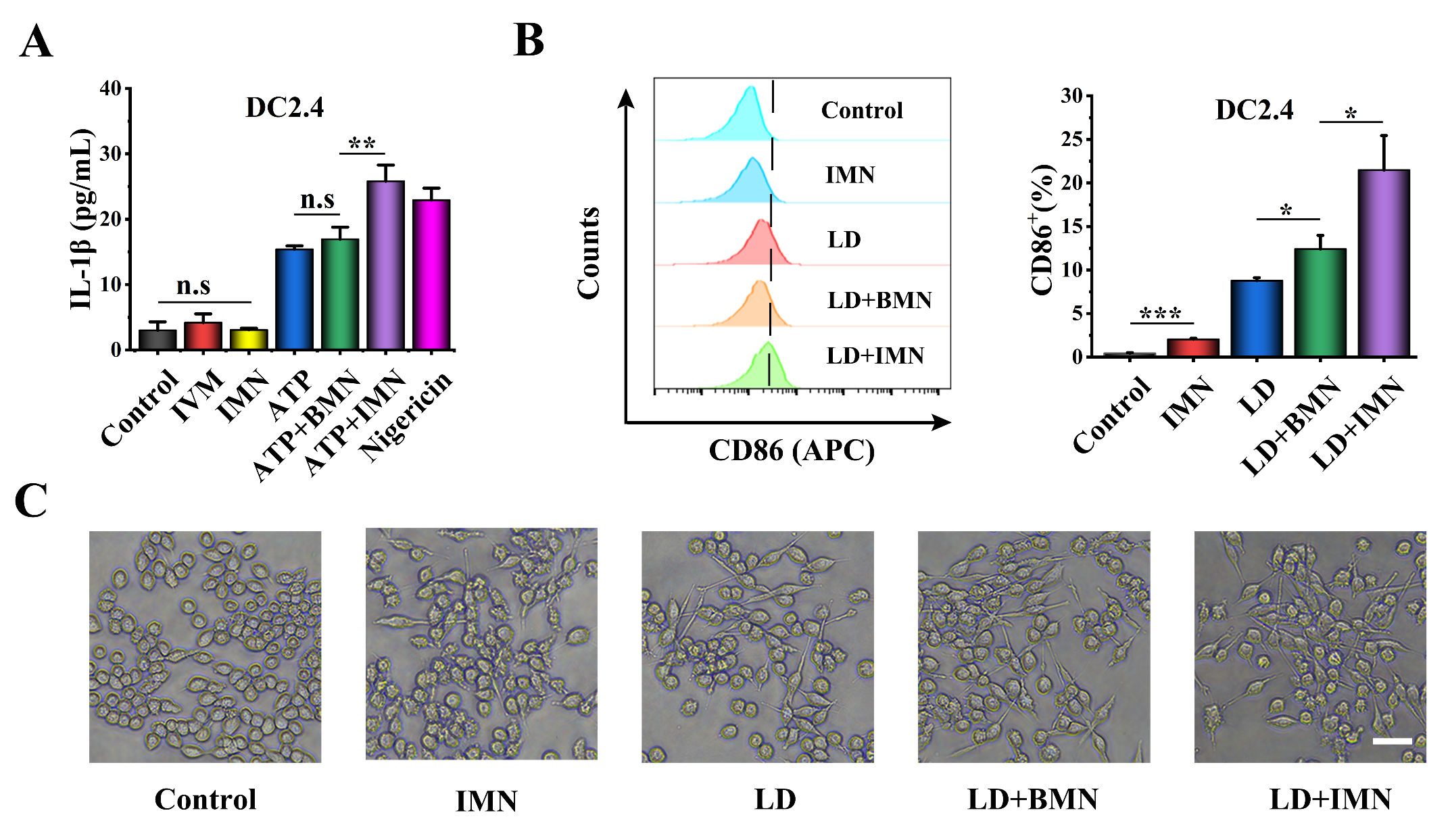


**Figure S6.** Activation of NLRP3 inflammasome in DC2.4 cells by ATP and IMN co-treatment. (**A**) Release of IL-1β from DC2.4 cells following various treatments. (n=3). (**B**) Flow cytometry analysis of DC2.4 cells co-cultured with supernatants from Hepa1-6 cells subjected to different interventions. (n=3). (**C**) Morphological changes in DC2.4 cells induced by supernatants from Hepa1-6 cells treated with the respective interventions. (Scale bar= 50 μm).


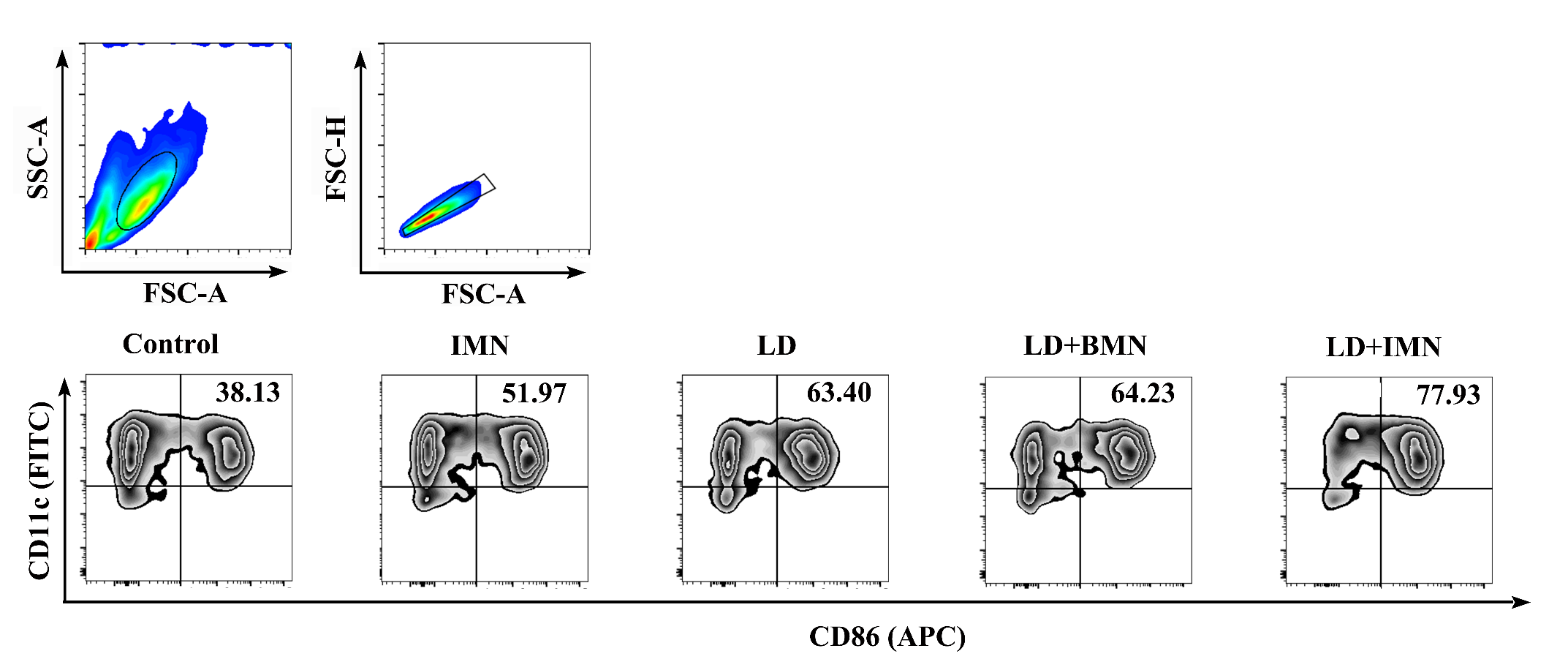


**Figure S7.**  Representative flow cytometry plots of BMDCs following different treatments (gated on CD11c^+^, and CD86^+^).


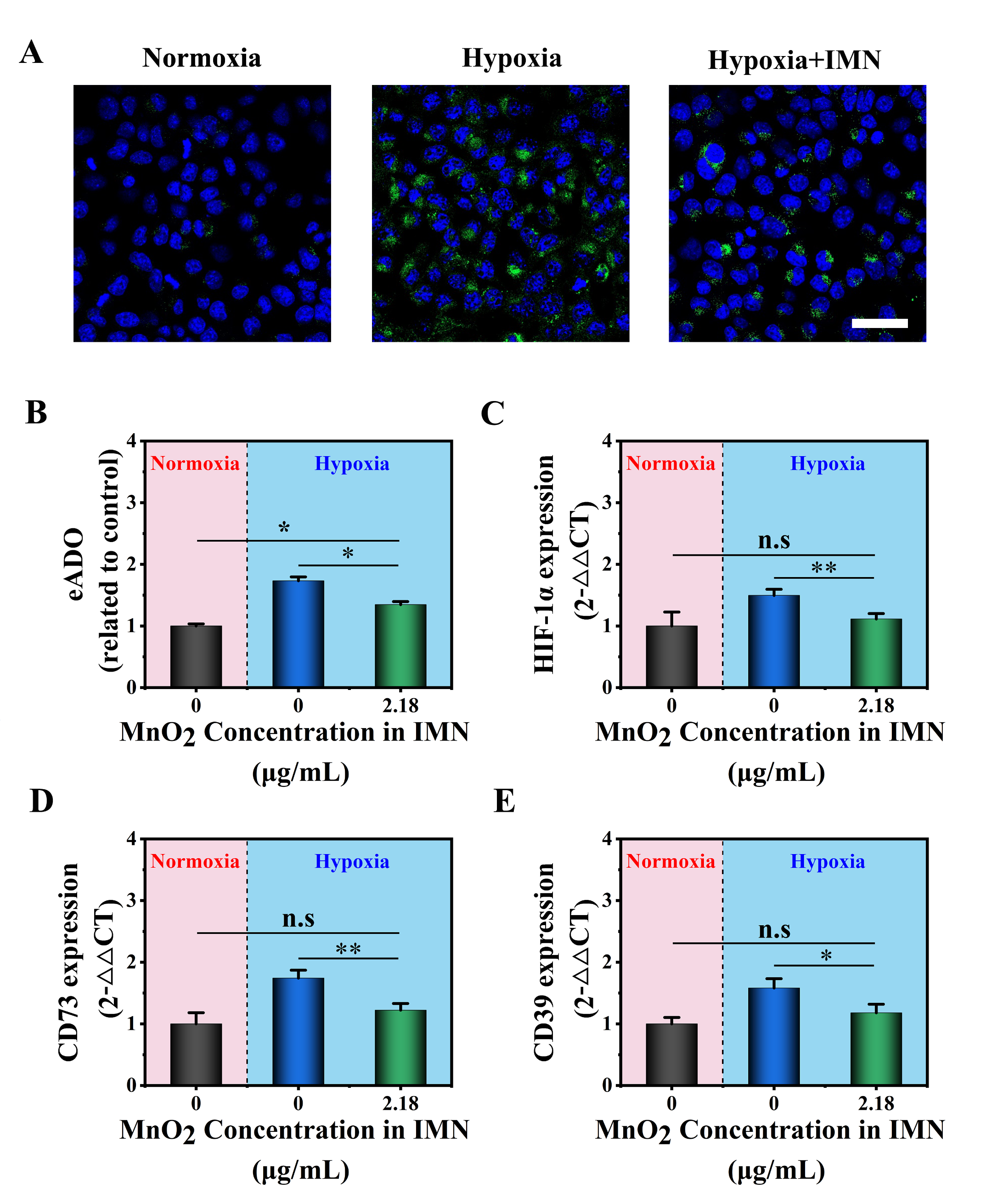


**Figure S8.** IMN alleviated hypoxia and modulated hypoxia-induced markers in Huh-7 cells. (**A**) Representative images of hypoxia detection in Huh-7 cells using Hypoxyprobe-1^TM^ staining under normoxic, hypoxic, and hypoxic+IMN conditions. Nuclei were stained blue with DAPI, while hypoxic cells appeared green. (n=3, Scale bar= 50 μm). (**B**) Quantitative assessment of eADO levels in cell supernatants after 24 hours of culture with or without IMN under normoxic or hypoxic conditions. (n=3). Expression levels of key molecular markers related to hypoxia-induced tumor aggression: (**C**) HIF1α, (**D**) CD73, and (**E**) CD39 in Huh-7 cells after 24 hours of incubation with varying concentrations of IMN under normoxic and hypoxic conditions. (n=3).


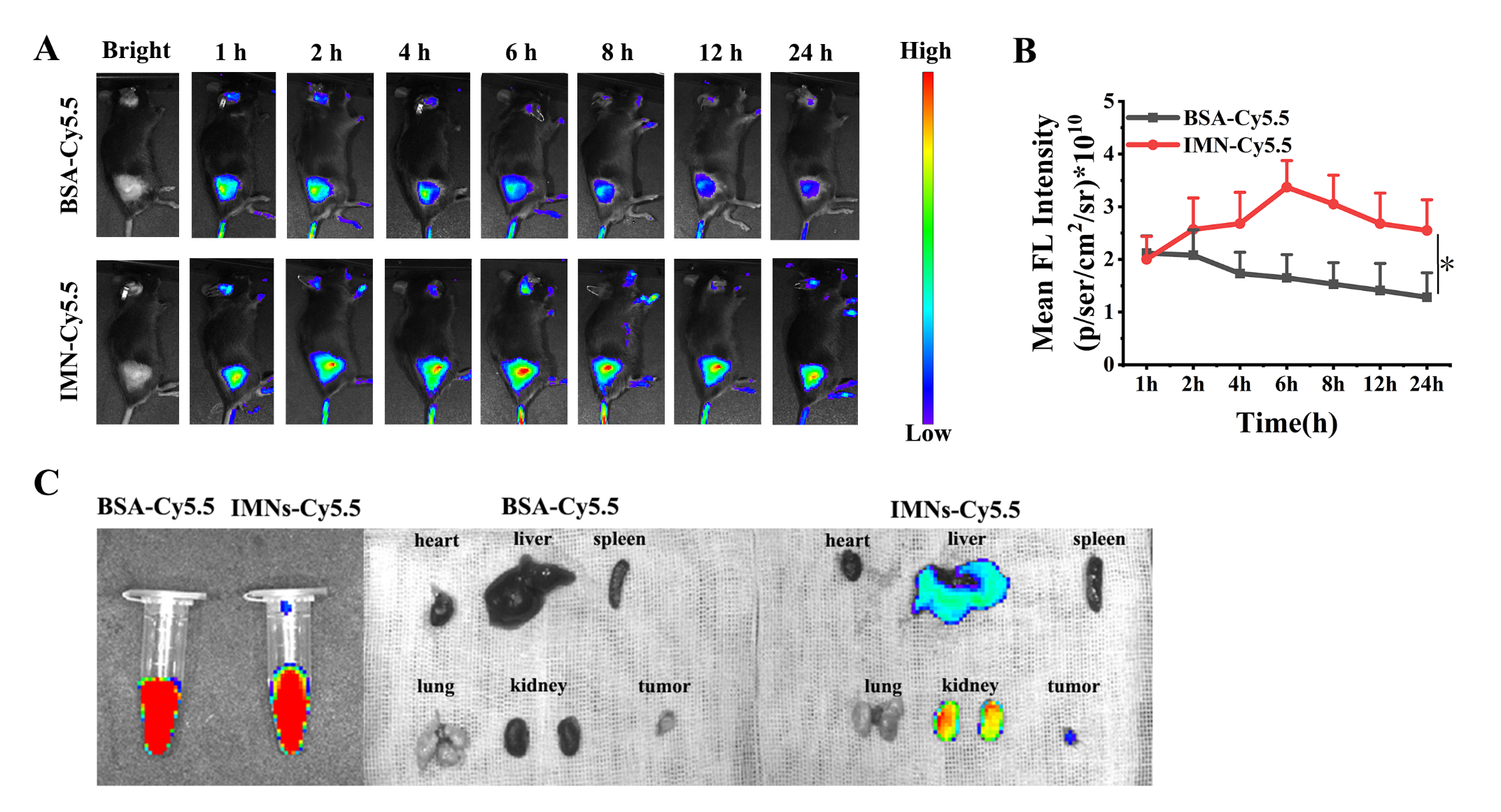


**Figure S9.**  In vivo biodistribution of IMN-Cy5.5 and BSA-Cy5.5 in Hepa1-6 HCC-bearing mice. (**A**) Representative in vivo fluorescence images of Hepa1-6 HCC-bearing mice at different time points after intravenous injection of IMN-Cy5.5 and BSA-Cy5.5. (**B**) Quantification of the average fluorescence intensity of tumors over time. (n=3). (**C**) The fluorescence intensity images of IMN-Cy5.5 and BSA-Cy5.5 for the intravenous injection (left panel). Quantification of the ex vivo fluorescence intensity of tumors and major organs from Hepa1-6 tumor-bearing mice 24 hours after injection of IMN-Cy5.5 and BSA-Cy5.5(right panel).


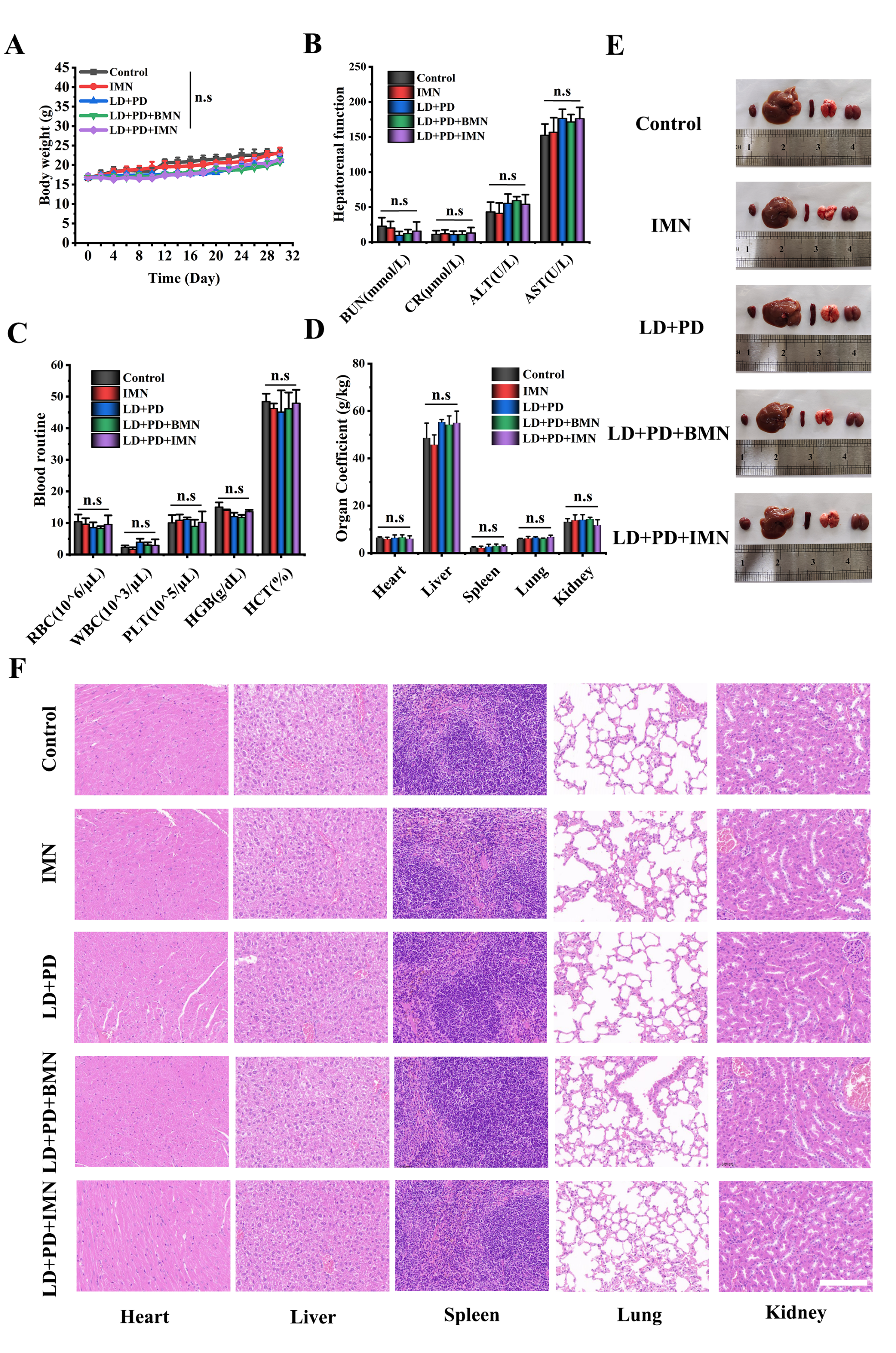


**Figure S10.**  Safety assessment of the antitumor therapy using LD+PD+IMN. (**A**) Body weight change over time during the experiment. (n=5). (**B**) Biochemical markers indicative of hepatorenal function, encompassing blood urea nitrogen (BUN), serum creatinine (CR), alanine aminotransferase (ALT), and aspartate aminotransferase (AST), assessed in mice following the experimental intervention. (n=3). (**C**) Quantitative analysis of essential hematological indices—namely, red blood cell count (RBC), white blood cell count (WBC), platelet count (PLT), hemoglobin concentration (HGB), and hematocrit (HCT), assessed in mice following the experimental intervention. (n=3). (**D**) Organ coefficients and (**E**) representative images of major organs (heart, liver, spleen, lung, and kidney) from mice after the experiment. (**F**) Histopathological examination of major organs (heart, liver, spleen, lung, and kidney) using hematoxylin-eosin staining. Scale bar= 100 µm.


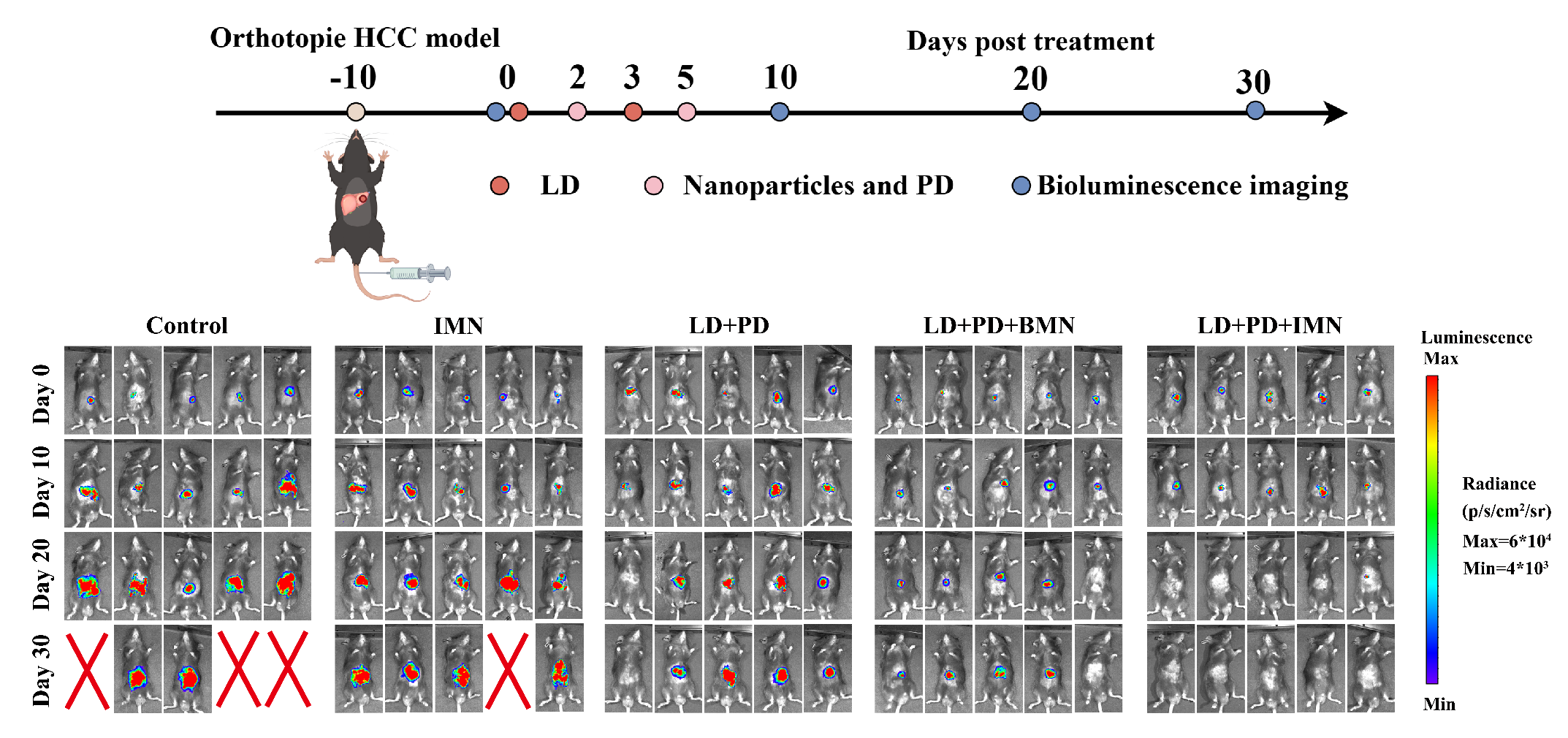


**Figure S11.** Monitoring of tumor burden in mice bearing orthotopic xenografts was conducted utilizing bioluminescence imaging. (n=5).


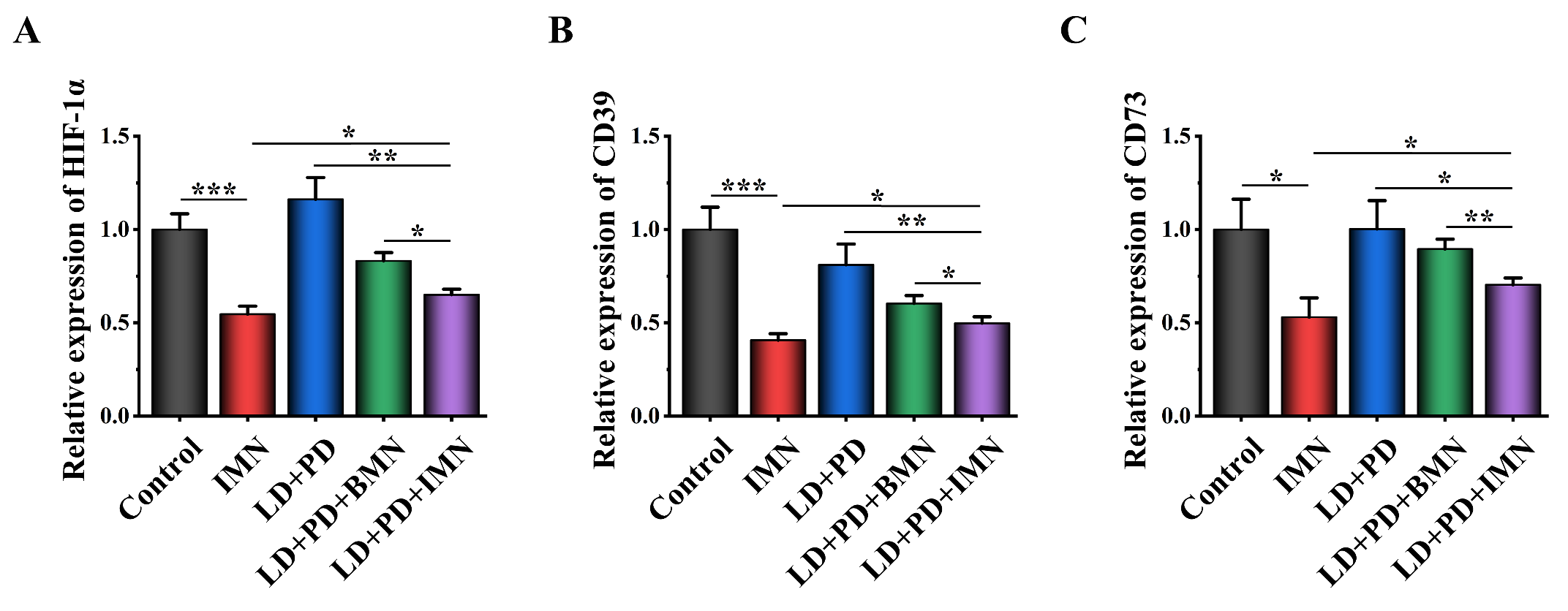


**Figure S12.** Densitometric analysis of Western blots was performed using ImageJ. Band intensities were quantified from three independent experiments and normalized to GAPDH (n=3).


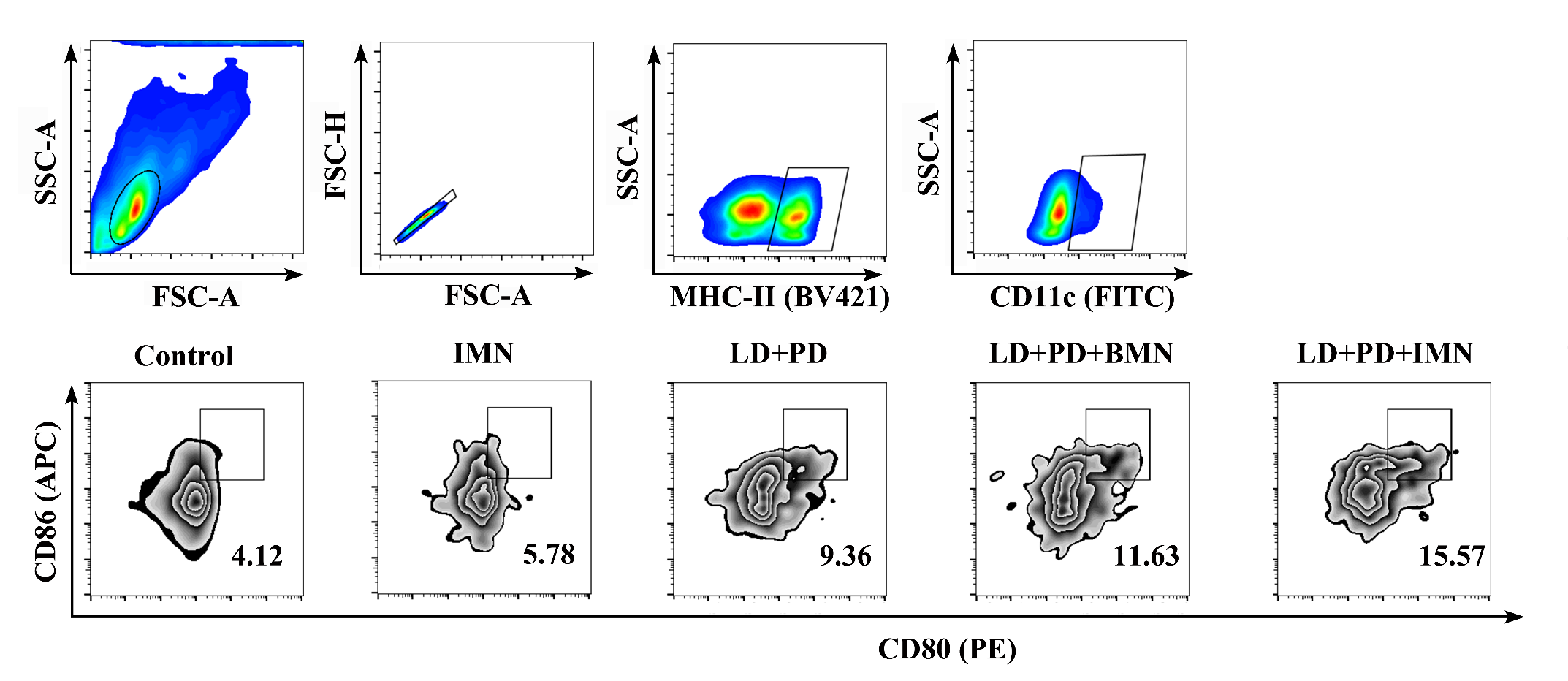


**Figure S13.** Representative flow cytometry plots of mature DCs in TDLNs at day 10 following different treatments (gated on MHC-II^+^, CD11c^+^, CD86^+^, and CD80^+^).


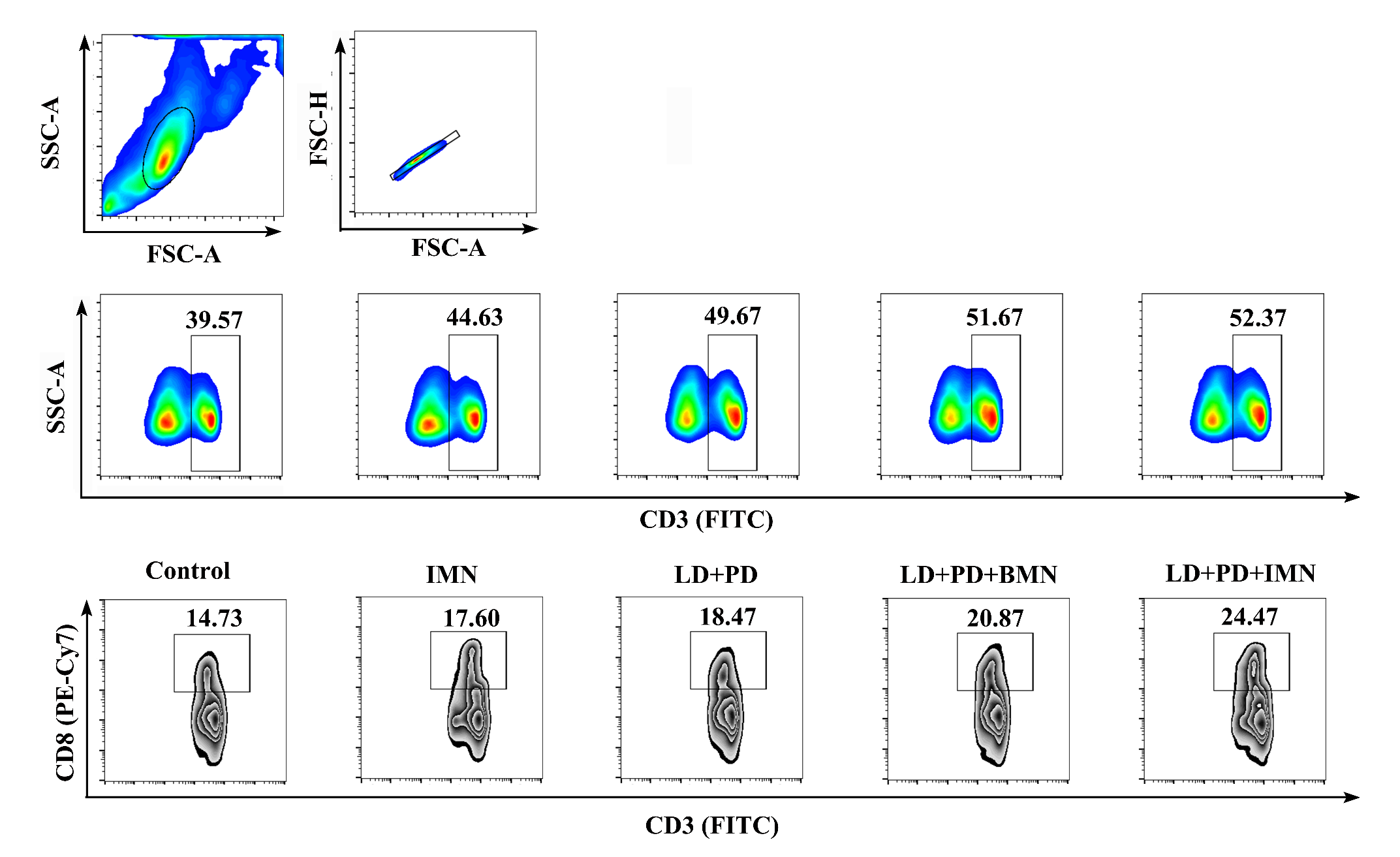


**Figure S14**. Representative flow cytometry plots of CD3^+^CD8^+^ T cells in spleens at day 10 following different treatments (gated on CD3^+^ and CD8^+^).


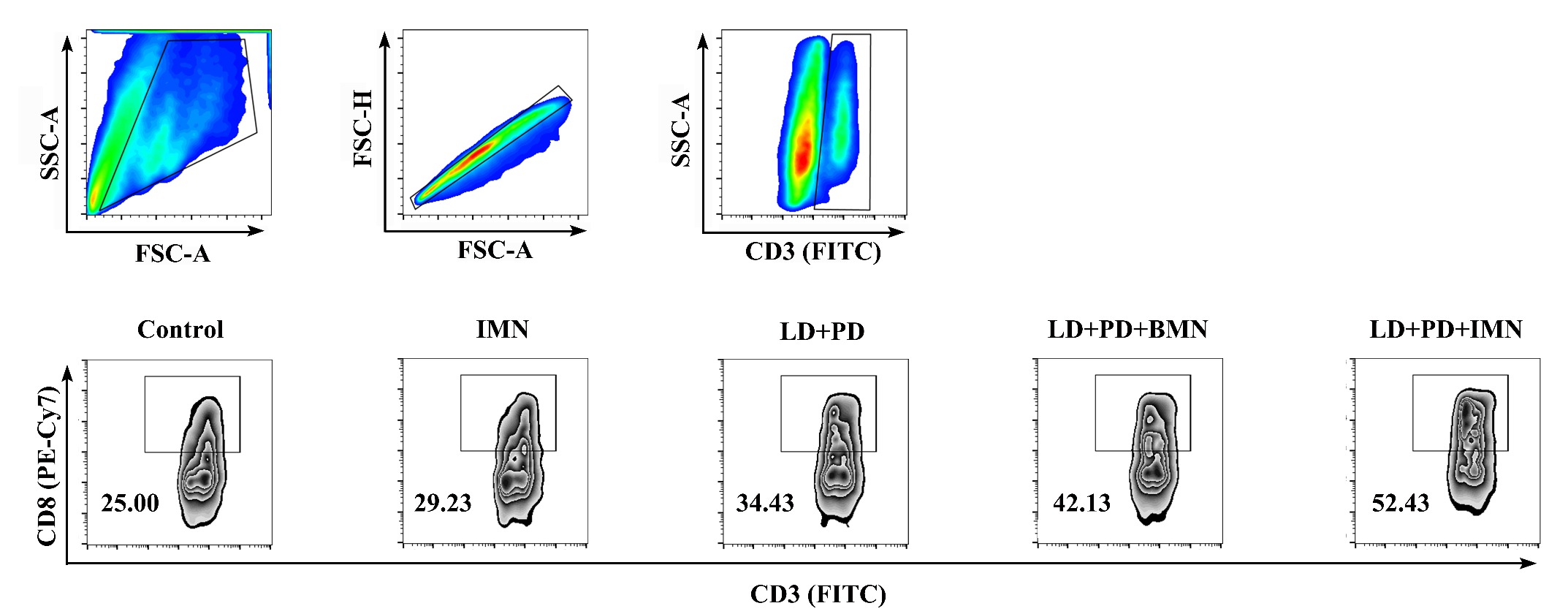


**Figure S15.**  Representative flow cytometry plots of CD3^+^CD8^+^ T cells in tumors at day 10 following different treatments (gated on CD3^+^ and CD8^+^).


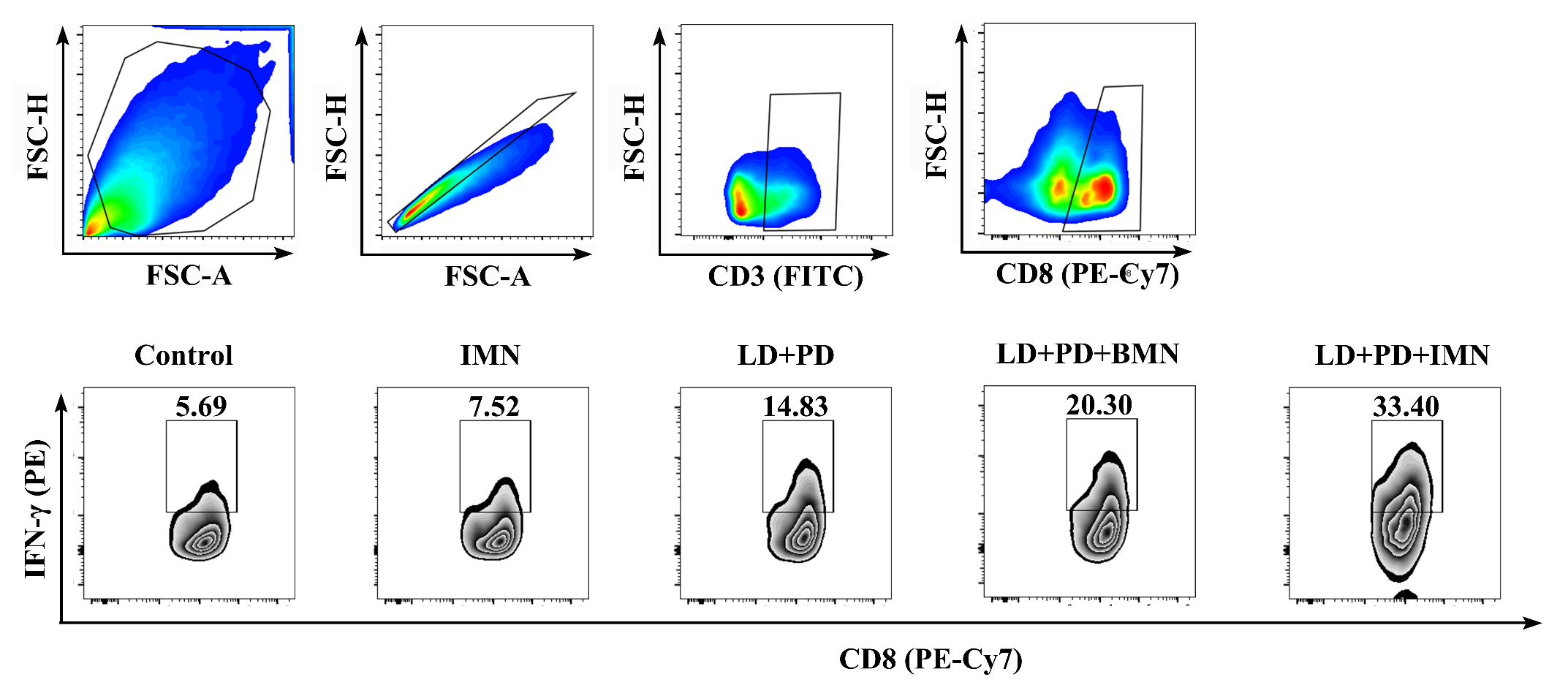


**Figure S16.**  Representative flow cytometry plots of IFN-γ^+^CD3^+^ CD8^+^ T cells in tumors at day 10 post-treatment. (gated on IFN-γ^+^, CD3^+^, and CD8^+^).


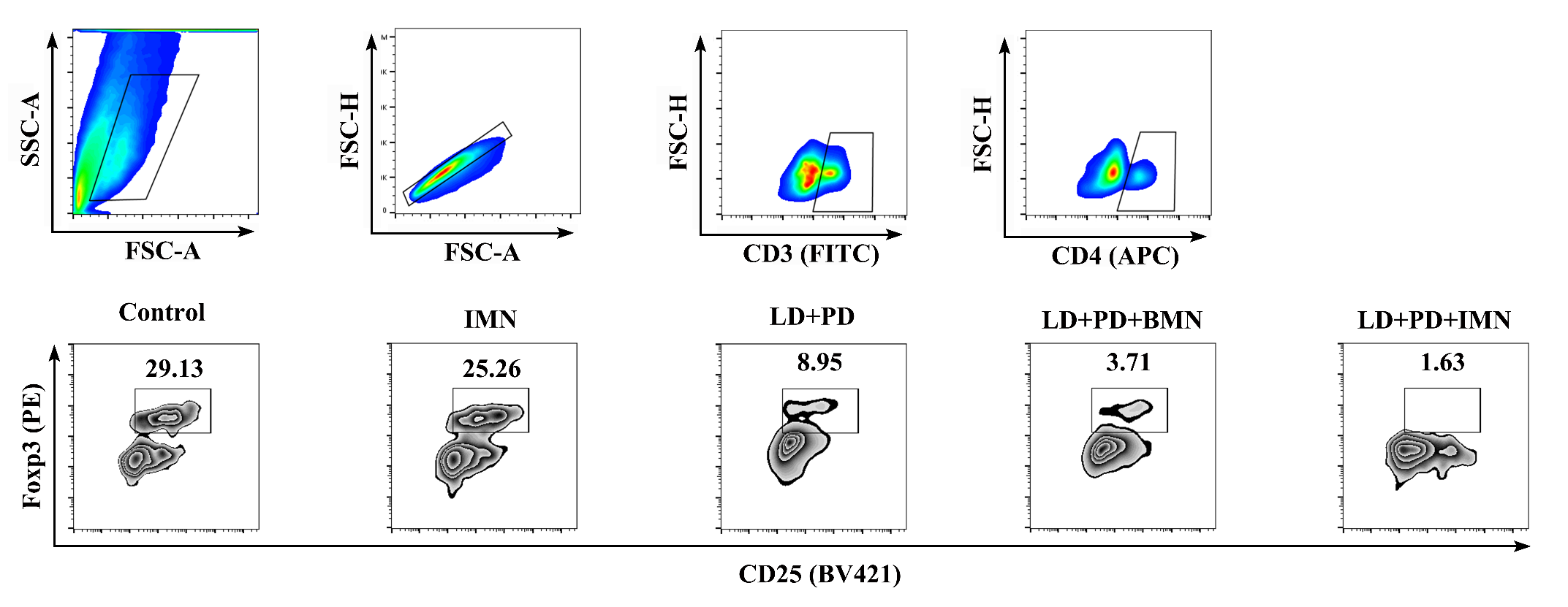


**Figure S17.**  Representative flow cytometry plots of Tregs cells in tumors at day 10 post-treatment. (gated on CD3^+^, CD4^+^, CD25^+^, and Foxp3^+^).


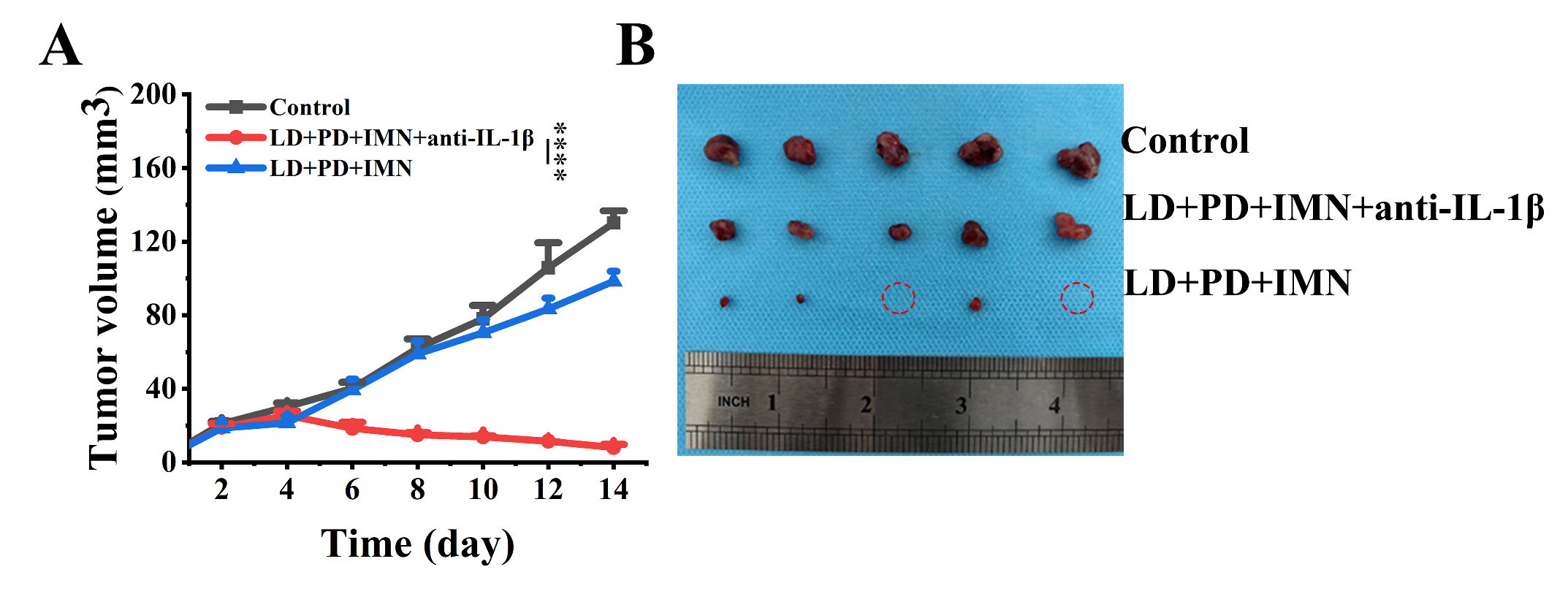


**Figure S18.**  Abrogation of antitumor efficacy by anti-IL-1β in combination with LD+PD+IMN (**A**) Tumor volume progression in the Hepa1-6 HCC-bearing C57BL/6J mouse model after treatment with saline, LD+PD+IMN+anti-IL-1β, or LD+PD+IMN. (n=5). (**B**) Tumor images from the same model after treatment with saline, LD+PD+IMN+anti-IL-1β, or LD+PD+IMN at the end of the experiment.

**Table S1. List of antibodies used for flow cytometry**

| **Antibody** | **Clone** | **Specificity** | **Source** | **Catalog** | **Used at** |
| --- | --- | --- | --- | --- | --- |
| CD16/32 | [S17011E](https://www.biolegend.com/en-gb/search-results?Clone=S17011E) | Mouse | Biolegend | 156604 | 1:200 |
| Mouse I-A/I-E | M5/114 | Mouse | BD Biosciences | 562564 | 1:200 |
| CD11c | HL3 | Mouse | BD Biosciences | 561045 | 1:200 |
| CD86 | GL1 | Mouse | BD Biosciences | 561964 | 1:200 |
| CD80 | 16-10A1 | Mouse | BD Biosciences | 561955 | 1:200 |
| CD3 | 145-2C11 | Mouse | BD Biosciences | 553062 | 1:200 |
| CD8 | 53-6.7 | Mouse | BD Biosciences | 561097 | 1:200 |
| CD4 | RM4-5 | Mouse | BD Biosciences | 553051 | 1:200 |
| CD25 | PC61 | Mouse | BD Biosciences | 562606 | 1:200 |
| Foxp3 | R16-715 | Mouse | BD Biosciences | 563101 | 1:100 |
| IFN-γ | W18272D | Mouse | Biolegend | 163504 | 1:200 |

**Table S2.** **Primer sequences for GAPDH, CD39, CD73 and HIF-1α used in RT-PCR test.**

| Hepa1-6 | | |
| --- | --- | --- |
| GAPDH | Forward | 5’-CATCACTGCCACCCAGAAGACTG-3’ |
|  | Reverse | 5’-ATGCCAGTGAGCTTCCCGTTCAG-3’ |
| CD39 | Forward | 5’-AGTTAGAGGAATGCCAAGTGAA-3’ |
|  | Reverse | 5’-CTCCTTTACTCCAGCGTAAGAT-3’ |
| CD73 | Forward | 5’-ATTCTGAGCGCAAACATTAAGG-3’ |
|  | Reverse | 5’-AGGGCGATGATCTTATTCACAT-3’ |
| HIF-1α | Forward | 5’-GAATGAAGTGCACCCTAACAAG-3’ |
|  | Reverse | 5’-GAGGAATGGGTTCACAAATCAG-3’ |
| Huh-7 | | |
| GAPDH | Forward | 5’-AGATCCCTCCAAAATCAAGTGG-3’ |
|  | Reverse | 5’-GGCAGAGATGATGACCCTTTT-3’ |
| CD39 | Forward | 5’-CTCAGGAAAAGGTGACTGAGAT-3’ |
|  | Reverse | 5’-CTCCTTTACTCCAGCGTAAGAT-3’ |
| CD73 | Forward | 5’-TGGTGGAGATGGGTTCCAGA-3’ |
|  | Reverse | 5’-CCGACCTTCAACTGCTGGAT-3’ |
| HIF-1α | Forward | 5’-TTTTGGCAGCAACGACACAG-3’ |
|  | Reverse | 5’-GTGCAGGGTCAGCACTACTT-3’ |

**Table S3.** **Characterization of nanoparticles (n=3)**

| Preparation | Diameter（nm） | PDI | Zeta potential  (mV) | EE(%) | LE(%) |
| --- | --- | --- | --- | --- | --- |
| BMN | 5.26±1.25 | 0.213±0.005 | -12.18±1.27 |  |  |
| IMN | 78.34±2.74 | 0.276±0.008 | -14.83±2.34 | 82.24±0.02 | 14.53±0.04 |
